# Transient Sleep Deprivation Induces Persistent Auditory Neuropathy via ROS‐Initiated Neuroinflammation and BK Channel Suppression

**DOI:** 10.1002/advs.77570

**Published:** 2026-09-08

**Authors:** Dan Chen, Ying Ma, Lixuan Huang, Xin Chen, Fanliang Kong, Zhenzhou Qian, Mingyue Guan, Xinlin Wang, Yanyan Yang, Shasha Zhang, Peng Li, Renjie Chai

**Affiliations:** ^1^ Department of Otorhinolaryngology The Third Affiliated Hospital of Sun Yat‐Sen University Guangzhou China; ^2^ Spine Surgery Department, Nantong First People's Hospital, State Key Laboratory of Digital Medical Engineering School of Life Sciences and Technology Advanced Institute For Life and Health Jiangsu Province High‐Tech Key Laboratory for Bio‐Medical Research Southeast University Nanjing China; ^3^ Southeast University Shenzhen Research Institute Shenzhen China; ^4^ Co‐Innovation Center of Neuroregeneration Nantong University Nantong China; ^5^ Department of Neurology Aerospace Center Hospital, School of Life Science Beijing Institute of Technology Beijing China

**Keywords:** auditory neuropathy, BK channels, cochlea, neuroinflammatory memory, sleep deprivation

## Abstract

The link between sleep disorders and sensorineural hearing loss (SNHL) is a critical clinical issue, yet the underlying mechanisms remain elusive. Here, we first establish a strong, dose‐dependent association between the severity of sleep disturbance and the degree of hearing loss in a clinical cohort of patients with sudden SNHL. To dissect causal mechanisms, we employ a mouse model of acute sleep deprivation (SD), demonstrating that SD causes a long‐lasting, high‐frequency hearing loss that, unlike restraint stress, cannot be attributed to generalized systemic stress. The core pathology is a tonotopically restricted cochlear synaptopathy, characterized by loss of hair cell ribbon synapses, initiated by transient oxidative stress and maintained by a chronic, self‐sustaining neuroinflammatory program, that drives persistent transcriptional suppression of large‐conductance calcium‐activated potassium (BK) channels. Tonotopic BK channels suppression is functionally validated by single‐cell electrophysiology and closely tracks frequency‐specific hearing loss. Critically, pharmacological intervention with the antioxidant N‐Acetyl‐L‐cysteine (NAC) or the BK channel opener NS1619 during the acute phase prevents auditory and synaptic deficits, establishing causal necessity for each pathway node. Together, these findings delineate a causally validated cascade from sleep loss to cochlear pathology, identifying a putative neuroinflammatory memory and BK channels as a therapeutic target for stress‐induced sensory neuropathy.

## Graphical abstract

Sleep disturbance severity closely tracks hearing loss in a clinical cohort, yet the mechanistic link remains unclear. Acute sleep deprivation is shown to trigger transient cochlear oxidative stress that switches into a self‐sustaining neuroinflammatory state, suppressing BK channels and causing irreversible synaptopathy. Early antioxidant or BK channel‐targeted therapy blocks this cascade, exposing druggable windows for preventing permanent hearing loss.

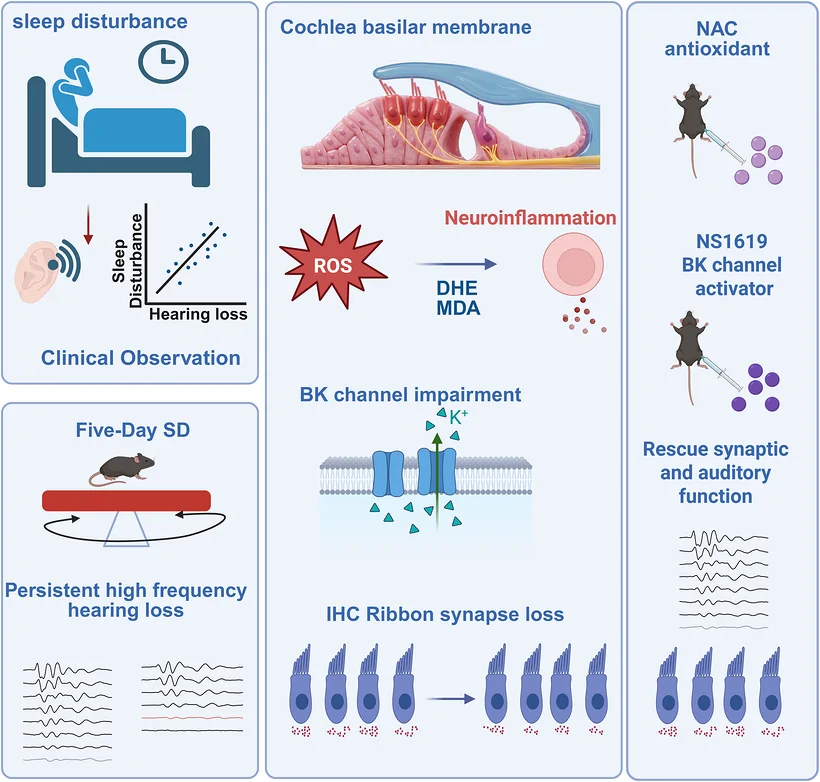

## 1 Introduction

**FIGURE 1:**
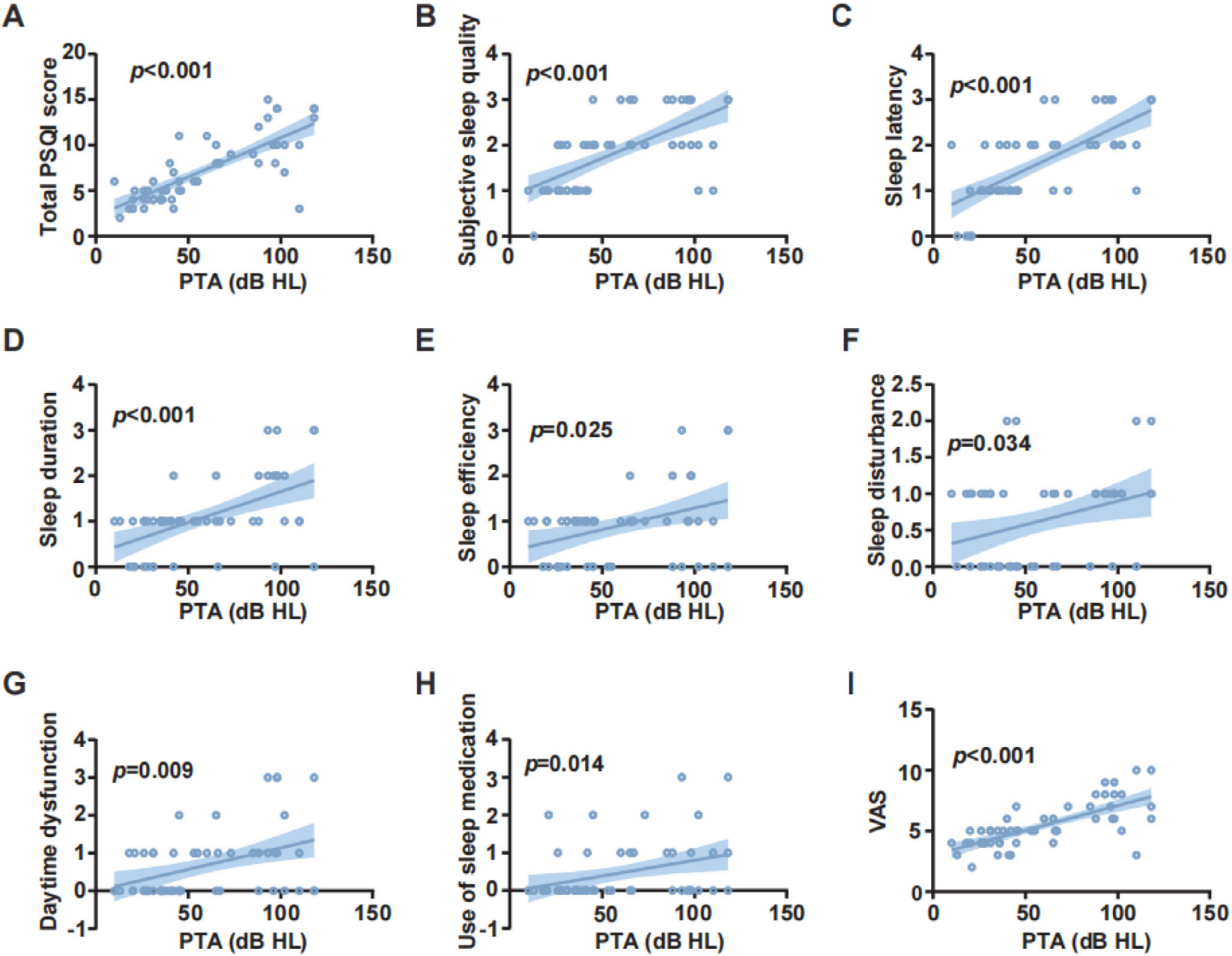
Sleep disturbance strongly correlates with the severity of hearing loss in patients with SSNHL. (A–I) Scatter plots showing the correlation between PTA thresholds and various metrics in a cohort of patients with SSNHL (n = 52). (A) Correlation with total PSQI score. (B–H) Correlations with individual PSQI components: (B) subjective sleep quality, (C) sleep latency, (D) sleep duration, (E) sleep efficiency, (F) sleep disturbance, (G) daytime dysfunction, and (H) use of sleep medication. (I) Correlation between PTA and subjective sleep quality assessed using a 10‐point sleep VAS, with higher scores indicating worse perceived sleep. Associations were evaluated using Spearman's rank correlation. The fitted line and 95% confidence interval are shown for visualization. *p*‐values were determined by Spearman's rank correlation analysis and are displayed in each plot.

Sensorineural hearing loss (SNHL) is a pervasive global health issue, yet for many forms, such as sudden SNHL (SSNHL), the underlying triggers and mechanisms remain elusive. A growing body of evidence suggests that systemic physiological states, including metabolic dysregulation, chronic inflammation, and stress can profoundly impact neural circuit integrity [1, 2]. Sleep disturbance, a ubiquitous and potent systemic stressor, is increasingly implicated in a range of neurological and sensory deficits [3, 4]. Indeed, the link between poor sleep and hearing disorders is not merely a distant epidemiological correlation but a pronounced clinical feature. Our own clinical data reveals a strong, dose‐dependent association between the severity of hearing loss in SSNHL patients and the degree of multidimensional sleep disturbance (Figure 1A–I, Table S1). This stark clinical reality establishes an urgent need to move beyond correlation and uncover the causal mechanisms by which sleep deprivation (SD) can drive auditory pathology.

A fundamental and unresolved challenge in neuropathology is to understand how a transient, body‐wide disruption can be converted into chronic, localized neural damage. Often, the initial systemic insult—such as the activation of the hypothalamic‐pituitary‐adrenal (HPA) axis and the subsequent surge in oxidative stress following SD—resolves within hours or days [5, 6]. Yet, the functional deficits in specific neural circuits can persist indefinitely or even progress. This critical disconnect implies the existence of a local, self‐perpetuating pathological mechanism that acts as a “memory” of the initial stressor, becoming uncoupled from the systemic trigger and taking on a life of its own within the affected tissue. However, the nature of this pathogenic bridge has remained a black box. Moreover, a major confounding factor in stress biology is distinguishing the specific neuropathological consequences of sleep loss from those induced by generalized systemic stress, a distinction rarely addressed in previous auditory research.

The auditory system provides an exceptional model to dismantle this process with unparalleled precision. The cochlea, the peripheral organ of hearing, contains a precise tonotopic map, with high frequencies processed at its base and low frequencies at its apex [7]. This anatomical organization, combined with its well‐defined circuitry of inner and outer hair cells (IHCs, OHCs), spiral ganglion neurons (SGNs), and supporting cells, allows for a high‐resolution analysis of circuit‐specific damage [8]. A primary site of vulnerability has been identified as the synapse between IHCs and SGNs. The loss of these connections without hair cell death, a condition termed cochlear synaptopathy or “hidden hearing loss”, is now recognized as a core driver of SNHL and perceptual difficulties, representing an ideal cellular endpoint to investigate subtle, stress‐induced neuropathology [9, 10].

While previous animal studies have confirmed that SD can induce or aggravate hearing loss [11, 12], they have not explained the paradox of persistence, nor have they definitively uncoupled the effects of sleep loss from general physiological stress. It remains entirely unknown how a transient systemic state leads to persistent, and often tonotopically‐specific, damage within the cochlea. Here, by combining in vivo functional testing with multi‐omic, electrophysiological, and pharmacological analyses in mice, we delineate a complete pathogenic cascade from systemic stress to molecular dysfunction. The study establishes that acute SD causes a persistent high‐frequency hearing loss, structurally underpinned by an irreversible, tonotopic loss of IHC synapses. Crucially, by employing a restraint stress paradigm, we demonstrate that this auditory neuropathy is uniquely attributable to SD rather than nonspecific systemic stress. A transient surge in cochlear oxidative stress is identified as the initiator of this damage. Subsequently, the pathology is shown to be maintained by a persistent, local inflammatory state that outlasts the systemic stressor. Through transcriptomic and patch‐clamp analyses, we link this chronic “neuroinflammatory memory” to the irreversible transcriptional suppression of large‐conductance calcium‐activated potassium (BK) channels at the cochlear base. Finally, targeted pharmacological interventions utilizing a reactive oxygen species (ROS) scavenger (N‐Acetyl‐L‐cysteine, NAC) or a specific BK channel activator (NS1619) successfully rescued the SD‐induced persistent cochlear ribbon synapse loss, establishing a definitive causal axis. The results thus reveal a ROS‐driven “neuroinflammatory memory” and subsequent BK channel suppression as a critical target for treating stress‐induced sensory disorders.

## 2 Results

### 2.1 Sleep Disturbance is a Core Clinical Feature and Correlates with the Severity of SNHL

To establish the clinical relevance of our study, we first investigated the association between sleep quality and hearing loss in a cohort of 52 patients with SSNHL and 36 healthy controls. The demographic and clinical characteristics are detailed in Table S1. As expected, SSNHL patients exhibited profound hearing impairment. However, a striking and consistent difference also emerged from the evaluation of sleep patterns using the validated Pittsburgh Sleep Quality Index (PSQI). The SSNHL patient group reported a mean total PSQI score of 7.94±4.39, a value significantly higher than the 4.03±1.83 reported by controls (*p* < 0.001) and well above the clinical cutoff for significant sleep disturbance. This was not a uniform impairment but a multidimensional collapse of sleep health, with patients exhibiting significant deficits across nearly all domains, including poorer subjective sleep quality, longer sleep latency, reduced sleep duration, and markedly lower sleep efficiency (Table S1). This paints a clear picture of chronic, disruptive sleep disorders as a prevalent comorbidity in SSNHL patient population.

To move beyond a simple group comparison and probe the depth of this association, we performed a Spearman's rank correlation analysis to determine if the severity of hearing loss correlated with the degree of sleep disturbance. As depicted in Figure 1A–I, this analysis revealed a striking and robust positive correlation between the Pure Tone Average (PTA), a measure of hearing loss severity, and the total PSQI score, which quantifies overall sleep disturbance (Figure 1A, *p* < 0.001). Specifically, hearing loss severity was significantly associated with poorer subjective sleep quality (Figure 1B, *p* < 0.001), increased sleep latency (Figure 1C, *p* < 0.001), and shorter sleep duration (Figure 1D, *p* < 0.001). Furthermore, patients with more severe hearing loss also reported lower sleep efficiency (Figure 1E, *p* = 0.025), more frequent sleep disturbances (Figure 1F, *p* = 0.034), greater daytime dysfunction (Figure 1G, *p* = 0.009), and a higher use of sleep medication (Figure 1H, *p* = 0.014). In addition to sleep‐related metrics, we observed a strong positive correlation between hearing loss severity (PTA) and the self‐reported severity of sleep disorder, as measured by a Visual Analog Scale (VAS) score (Figure 1I, *p* < 0.001). Taken together, these clinical data demonstrate a powerful, dose‐dependent association between the degree of auditory dysfunction and the pervasive nature of sleep disturbance, providing a compelling rationale for a mechanistic investigation into a potential causal link.

### 2.2 SD in Mice Induces a Persistent, Tonotopically‐Specific Auditory Neuropathy

**FIGURE 2:**
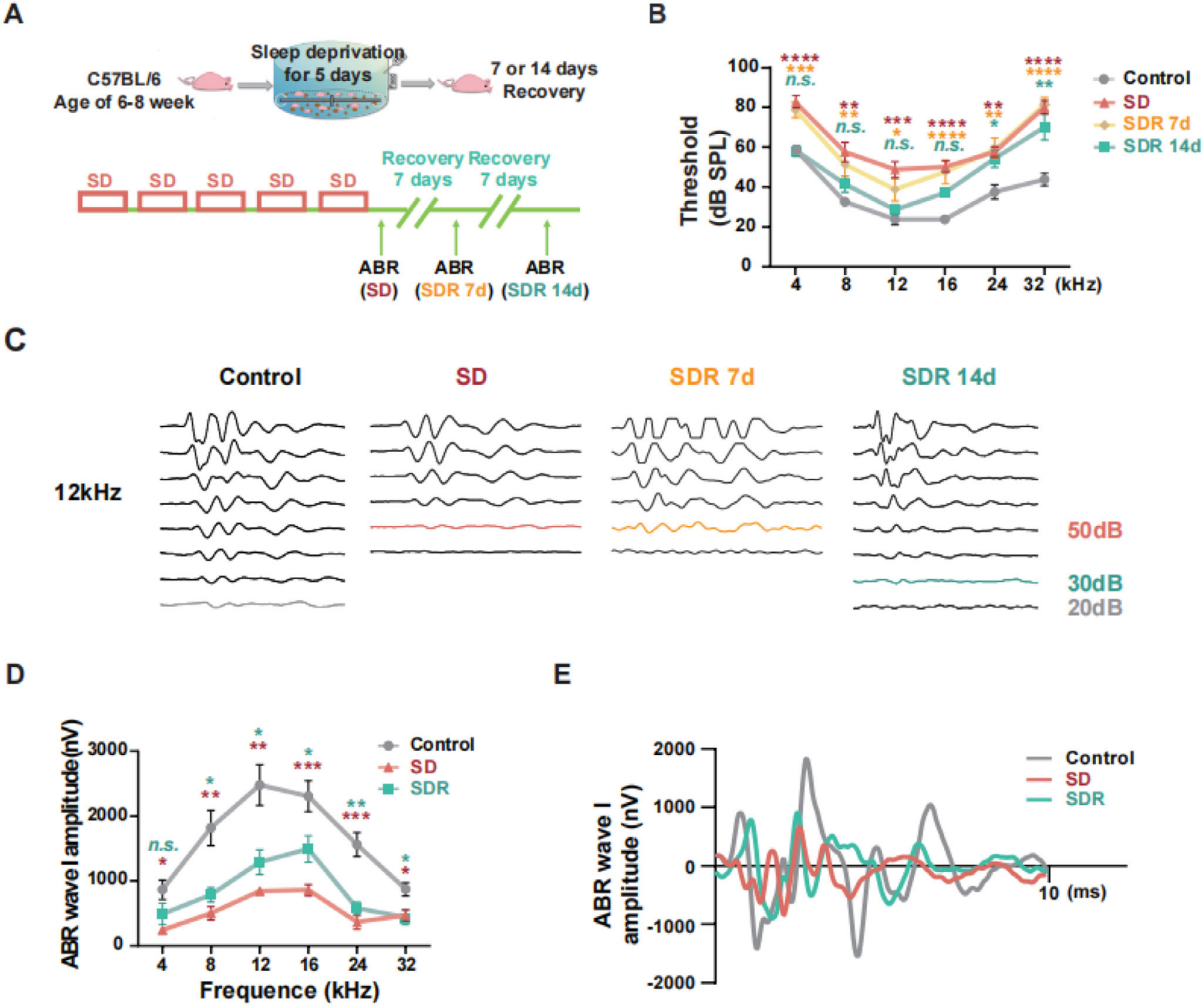
SD induces a partially irreversible, high‐frequency SNHL and persistent auditory neuropathy. (A) Schematic diagram of the experimental timeline, illustrating the 5‐day SD protocol followed by 7‐day (SDR 7d) or 14‐day (SDR 14d) recovery periods before analysis. (B) ABR thresholds (in dB SPL) across frequencies from 4 to 32 kHz for all Control, SD, SDR 7d and SDR 14d mice. (C) Representative ABR waveforms recorded at 12 kHz from Control, SD, SDR 7d and SDR 14d mice. (D) ABR wave I amplitude (in nV) at different frequencies for Control, SD, and SDR 14d groups. (E) Representative ABR wave I waveforms recorded at a supra‐threshold stimulus (90 dB SPL) at 12 kHz. Data are presented as mean ± SD. Statistical significance was determined by two‐way ANOVA with Tukey's multiple comparisons test. *n.s*., not significant; ^*^ *p* < 0.05, ^**^ *p* < 0.01, ^***^ *p* < 0.001, ^****^ *p* < 0.0001. n = 8 mice per group.

To test the link between sleep disturbance and hearing loss, we utilized a mouse model based on the protocol shown in Figure 2A and Figure S1A, subjecting mice to a consecutive 5‐day acute SD followed by a 7‐ or 14‐day recovery period. The protocol induced an expected physiological stress response, manifested as a transient decrease in body weight during the 5‐day SD period. This body weight quickly normalized and was indistinguishable from controls upon cessation of the protocol (Figure S1B), indicating that the subsequent persistent auditory pathology is not a consequence of a lasting systemic metabolic deficit but rather a more specific downstream consequence of the initial stressor.

The SD protocol had profound effects on the auditory system. Auditory brainstem response (ABR) recordings revealed a severe, pan‐frequency hearing impairment immediately after SD, with thresholds elevated by 30–40 dB (Figure 2B). While partial recovery was observed after 7 days, a more stable phenotype was present after 14 days. While thresholds at low‐to‐mid frequencies returned to baseline, a significant and persistent deficit remained at high frequencies (24 and 32 kHz) (Figure 2B,C and Figure S1C). We therefore selected the 14‐day time point (SDR 14d) for all subsequent analyses.

To dissect the nature of this hearing loss, we analyzed the ABR wave I amplitude, which reflects auditory nerve function. SD caused a marked reduction in wave I amplitude across all frequencies, a deficit that persisted in the SDR group, particularly at the high frequencies where threshold shifts remained (Figure 2D and Figure S1D). Representative ABR waveforms provided a clear qualitative illustration of this auditory neuropathy; responses in SD mice were severely attenuated and remained compromised at high frequencies even after recovery (Figure 2E). Together, these results demonstrate that acute SD induces a partially irreversible, high‐frequency auditory neuropathy in mice, establishing a robust preclinical model to investigate the cellular and molecular mechanisms underlying the clinically observed link between hearing loss and sleep disorders.

### 2.3 The Lasting Neuropathy is Caused by Cochlear Ribbon Synapse Loss, Not Hair Cell Loss

**FIGURE 3:**
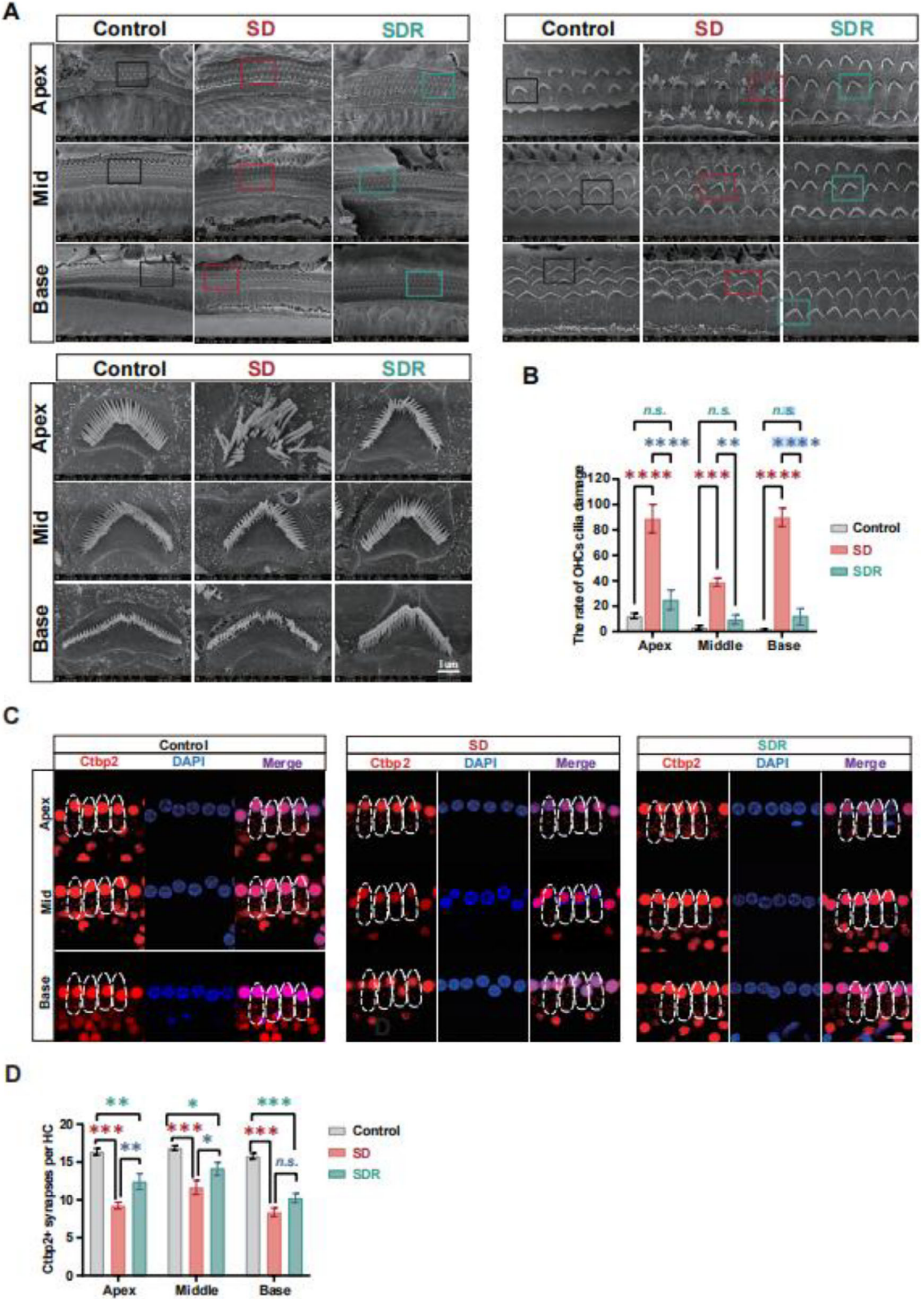
SD induces transient OHC stereocilia damage and persistent, tonotopic cochlear ribbon synapse loss. (A) Representative SEM images of the cochlear sensory epithelium from Control, SD, and SDR mice at the apex, middle, and base. Scale bar, 1 µm. (B) Quantification of the percentage of OHCs exhibiting damaged or disarrayed stereocilia bundles across the different cochlear regions (n = 5 mice per group). (C) Representative confocal images of IHC ribbon synapses from the apex, middle, and base. Cochlear sections were immunostained for the presynaptic ribbon protein Ctbp2 (red) and the nuclear marker DAPI (blue). IHCs are outlined with dashed white lines to highlight the reduction of synaptic puncta in the SD group and the incomplete recovery in the SDR group, particularly in the base. (D) Quantification of the average number of Ctbp2+ ribbon synapses per IHC across the three cochlear regions (n = 5 mice per group). Data in (B) and (D) are presented as mean ± SEM. Statistical significance was determined by one‐way ANOVA followed by Tukey's post–hoc test. ^****^ *p* < 0.0001, ^***^ *p* < 0.001, ^**^ *p* < 0.01, ^*^ *p* < 0.05; *n.s*., not significant.

To identify the cellular basis for the observed auditory neuropathy, we first assessed the structural integrity of the cochlear sensory epithelium. Immunostaining for Myosin7a revealed no significant loss of either IHCs or OHCs in any cochlear region of experimental group (Figure S2A–C). This critical finding demonstrates that the hearing loss is not caused by overt sensory cell death. Further examination by scanning electron microscopy (SEM) revealed severe disarray and fusion of OHC stereocilia bundles immediately after SD (Figure 3A). Remarkably, however, this pathology was completely resolved following the recovery period, with stereocilia morphology in the SDR group returning to a state indistinguishable from controls (Figure 3A,B). While the acute stereocilia damage likely contributes to the initial pan‐frequency hearing loss, its recovery indicates that it is not the cause of the persistent high‐frequency deficit.

Given the pronounced and persistent reduction in the ABR wave I amplitude, we next investigated the integrity of the ribbon synapses connecting IHCs to SGNs. Immunostaining for the presynaptic ribbon protein Ctbp2 revealed a dramatic reduction in synaptic puncta at the base of IHCs in the SD group across all cochlear regions (Figure 3C,D). Critically, following recovery, synaptic counts were only partially restored in the low‐frequency apex and middle turns and remained significantly reduced in the high‐frequency basal turn. This tonotopic pattern of irreversible synaptic loss provides a direct structural correlate for the persistent high‐frequency hearing deficit. To confirm the lesion was restricted to the synapse, we stained for the neuronal marker Tuj1 and found no loss of SGN peripheral fibers, indicating that the auditory nerve itself remained intact (Figure S2D,E).

To further determine whether the auditory phenotype induced by SD was merely a nonspecific consequence of systemic stress, a separate cohort of mice was subjected to restraint stress for 6 h per day for five consecutive days (Figure S3A). Restraint stress significantly elevated serum corticosterone levels, confirming robust activation of the physiological stress response (Figure S3B). Nevertheless, ABR thresholds remained unchanged across all tested frequencies following restraint stress (Figure S3C). Moreover, the numbers of CtBP2+ ribbon synapses per IHC were comparable between restraint‐stressed and control mice in the apical, middle, and basal cochlear turns (Figure S3D,E). These findings indicate that, under the present experimental conditions, a generalized restraint‐induced stress response alone is insufficient to reproduce the auditory dysfunction and cochlear ribbon synapse loss induced by SD. Together, these findings demonstrate that acute SD induces persistent, predominantly high‐frequency auditory dysfunction accompanied by cochlear ribbon synapse loss, supporting cochlear ribbon synapse loss as a key structural substrate of the SD‐induced auditory phenotype. Importantly, the absence of such pathology in the restraint stress cohort confirms that this auditory phenotype is a specific consequence of sleep loss, rather than a mere byproduct of generalized systemic stress.

### 2.4 Persistent Cochlear Ribbon Synapse Loss is Initiated by a Massive but Transient Wave of Oxidative Stress

**FIGURE 4:**
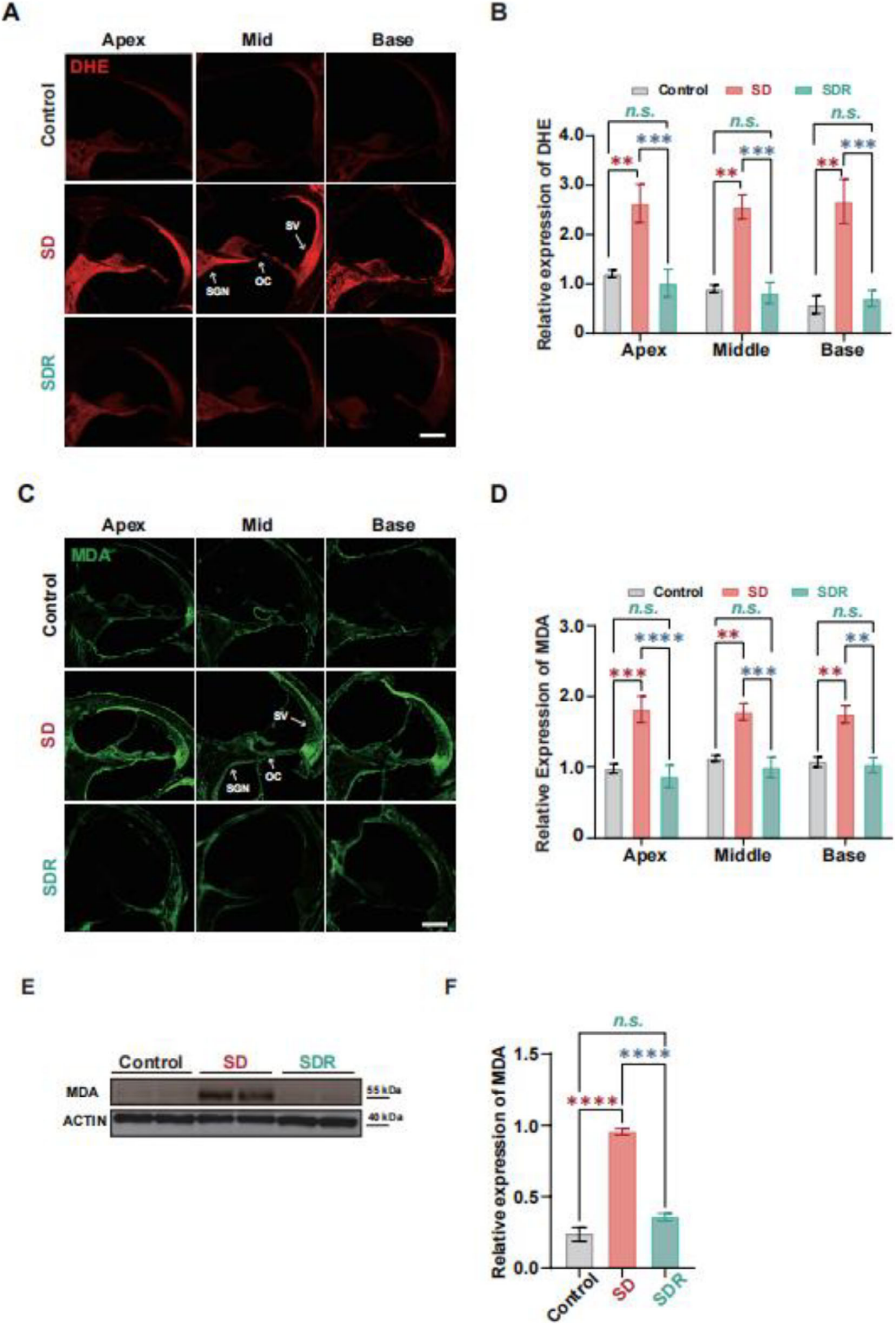
SD Induces a Transient Oxidative Insult that Triggers Persistent Cochlear Ribbon Synapse Loss. (A) Representative immunofluorescence images of cochlear cross‐sections stained for ROS with DHE (red). Regions shown include the SGN, OC, and SV. Scale bar, 50 µm. (B) Quantification of relative DHE fluorescence intensity in the SGN, OC, and SV regions. (C) Representative immunofluorescence images of cochlear cross‐sections stained for the lipid peroxidation marker MDA (green). Regions shown include the SGN, OC, and SV. Scale bar, 50 µm. (D) Quantification of relative MDA fluorescence intensity in the SGN, OC, and SV regions. (E) Representative western blot for MDA‐modified proteins and β‐actin (loading control) in whole cochlear lysates from Control, SD, and SDR mice. (F) Quantification of relative MDA expression from western blots, normalized to β‐actin. Data are presented as mean ± SEM. Statistical significance was determined by one‐way ANOVA with Tukey's post–hoc test. *n.s*., not significant, ^****^ *p* < 0.0001, ^***^ *p* < 0.001, ^**^ *p* < 0.01. (spiral ganglion neurons, SGN; organ of Corti, OC; stria vascularis, SV).

We next sought to identify the initial trigger for this synaptic damage. As SD is a potent systemic stressor, we hypothesized that the pathology is initiated by a consequential wave of oxidative stress. We first measured ROS using dihydroethidium (DHE) staining and observed a widespread and intense increase in DHE fluorescence in the SD group across SGNs, the organ of Corti (OC), and the stria vascularis (SV) (Figure 4A,B). Consistent with this, the lipid peroxidation marker malondialdehyde (MDA) was also markedly elevated, confirming significant oxidative damage (Figure 4C,D). This was further validated by western blot analysis of whole cochlear lysates, which showed a significant upregulation of MDA‐modified proteins in the SD group (Figure 4E,F).

The most striking finding, however, was the complete resolution of this oxidative stress state during the recovery period. In the SDR group, which still exhibited persistent cochlear ribbon synapse loss, both DHE fluorescence and MDA levels—measured by immunofluorescence and western blotting—were indistinguishable from those of control animals (Figure 4A–F). This establishes a critical temporal disconnect: a severe but transient insult (including both oxidative stress and stereocilia damage) precedes a persistent and localized auditory neuropathy. Therefore, while acute oxidative stress acts as a potent initiator of cochlear injury, it cannot, by itself, account for the chronic cochlear ribbon synapse loss. This strongly suggests that the initial oxidative wave must ignite a more durable, self‐perpetuating pathological process within the cochlea that is responsible for maintaining the long‐term damage.

### 2.5 SD Ignites a Sustained Neuroinflammatory Transcriptome in the Cochlea

**FIGURE 5:**
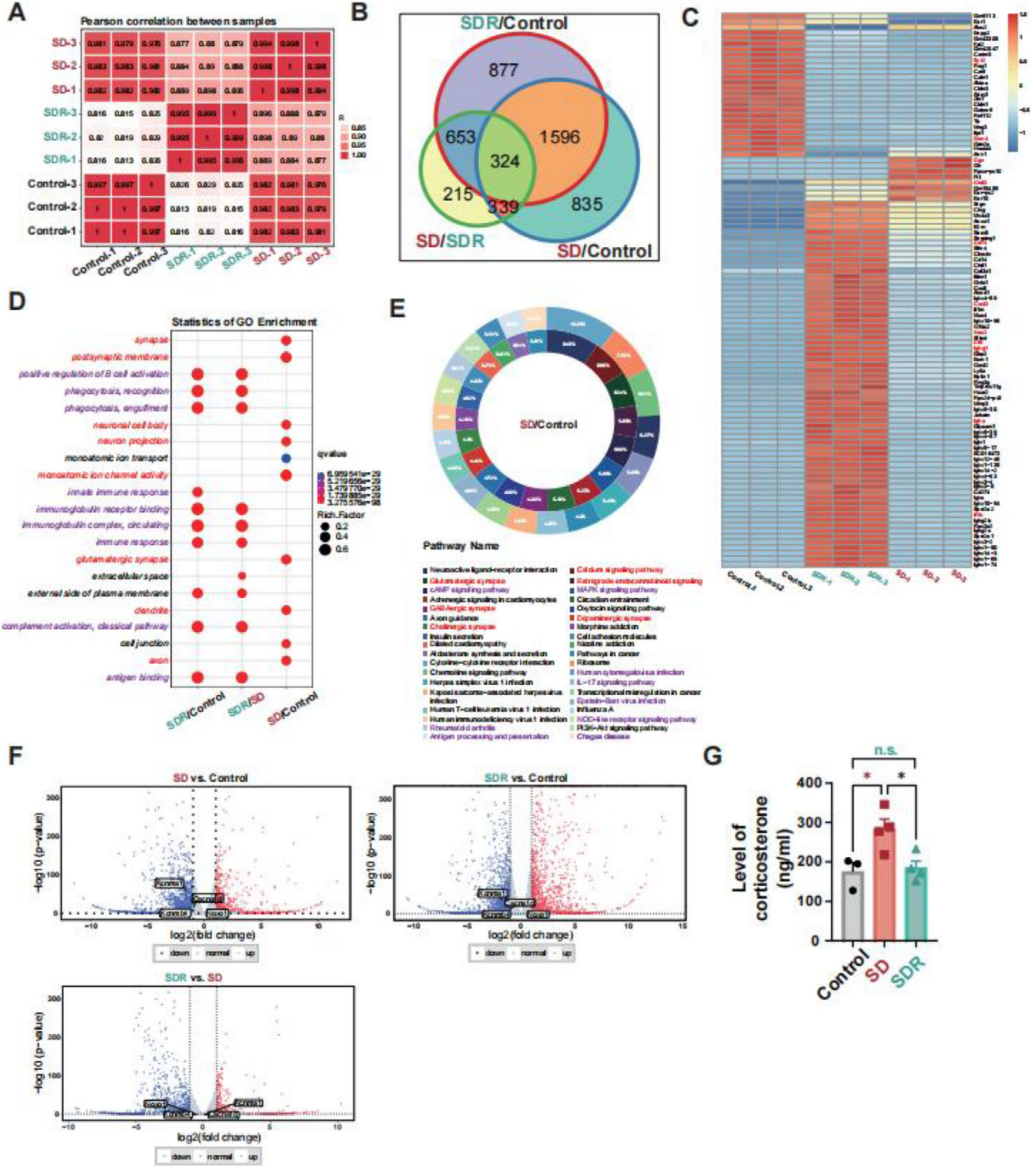
SD ignites a persistent neuroinflammatory transcriptome in the cochlea. (A) Pearson correlation heatmap of RNA‐sequencing data from whole cochleae, demonstrating distinct transcriptional profiles for each experimental group (Control, SD, SDR) and the failure of the SDR group to revert to the control state. (B) Venn diagram illustrating the overlap of DEGs between key comparisons. (C) Heatmap of the most significantly dysregulated genes. Genes are clustered by expression pattern. (D, E) GO (D) and KEGG pathway (E) enrichment analyses. (F) Volcano plot showing differentially expressed genes between groups. (G) Quantification of serum corticosterone levels. Data are presented as mean ± SEM. Statistical significance was determined by one‐way ANOVA with Tukey's multiple comparisons test (*n.s*., not significant; ^*^ *p* < 0.05). For RNA‐seq (A‐F), n = 3 mice per group. For corticosterone (G), n = 3 mice for Control, n = 4 mice for SD and SDR group.

If oxidative stress resolves, what mechanism sustains the cochlear pathology? To answer this, we performed RNA‐sequencing on cochleae from Control, SD, and SDR mice. Comprehensive RNA‐seq analysis of cochlear tissues revealed distinctive and persistent transcriptional changes following SD and subsequent 14‐day recovery. Principal component analysis (PCA) of the transcriptomic profiles revealed that the three experimental groups clustered distinctly, indicating substantial global gene expression alterations induced by SD and a unique, partially restored transcriptional state following recovery (Figure S4C). Furthermore, as depicted in Figure 5A, Pearson correlation analysis demonstrated high reproducibility among replicates, and hierarchical clustering robustly distinguished the Control, SD, and SDR groups based on their gene expression profiles. While SD induced 835 unique differentially expressed genes (DEGs), a core of 339 DEGs remained dysregulated after 14 days of recovery, indicating a lasting molecular scar (Figure 5B).

A heatmap of the most significant DEGs provided a detailed view of these transcriptional shifts (Figure 5C). The SD group was characterized by upregulation of acute stress‐related genes (e.g., *Cga*, *Chil3*). In contrast, the SDR group exhibited a profound and sustained upregulation of genes associated with a chronic immune and inflammatory response, including immunoglobulins (*Ighg1*, *Igha*), complement factors (*Cfb*), cytokines (*Il1b*), chemokines (*Cxcl3*), and key inflammatory mediators (*Nos2*, *Cd74*). Concurrently, genes critical for synaptic function (*Syt2*, *Grm4*) and ion homeostasis (*Kcnma1*, *Cacna1d*) were downregulated and failed to recover (Figure 5C,F). Gene Ontology (GO) and KEGG analysis confirmed that the cochlear pathology shifts from an acute response involving “neuroactive ligand‐receptor interaction” and “glutamatergic synapse” to a chronic state dominated by immune pathways like “cytokine‐cytokine receptor interaction” (Figure 5D,E).

This local inflammatory state persisted despite the normalization of the systemic stress response, as evidenced by serum corticosterone levels which were elevated in the SD group but returned to baseline in the SDR group (Figure 5G). These data demonstrate that an acute systemic stressor ignites a self‐sustaining, chronic neuroinflammatory program within the cochlea, which becomes the primary driver of persistent molecular and cellular damage.

### 2.6 Persistent Inflammation Drives Irreversible, Tonotopic Suppression of BK Channels

To identify a key molecular effector linking the sustained neuroinflammatory state to the auditory deficit, we mined our transcriptomic dataset for ion channel dysregulation. A volcano plot analysis of differentially expressed genes between the SD and control groups highlighted Kcnma1, which encodes the pore‐forming α‐subunit of the large‐conductance calcium‐activated potassium (BK) channel, as a highly significant SD‐responsive gene (Figure S4B). Given the physiological importance of both BK and small‐conductance calcium‐activated potassium (SK) channels in hair cell function, we systematically compared their expression patterns to determine the primary target of SD‐induced damage. A targeted heatmap showing the expression patterns of BK channel‐related genes (*Kcnma1*, *Kcnmb1*, and *Kcnmb2*) and SK channel‐related genes (*Kcnn1*, *Kcnn2*, and *Kcnn3*) across control, SD, and recovery groups revealed a striking divergence (Figure S4A). Compared with SK channel genes, which remained relatively stable, *Kcnma1* exhibited a much more prominent SD‐associated alteration and showed a tendency toward partial, yet incomplete, restoration after recovery. This robust and specific transcriptional vulnerability supported the prioritization of the BK channel as a candidate molecular target for further investigation.

**FIGURE 6:**
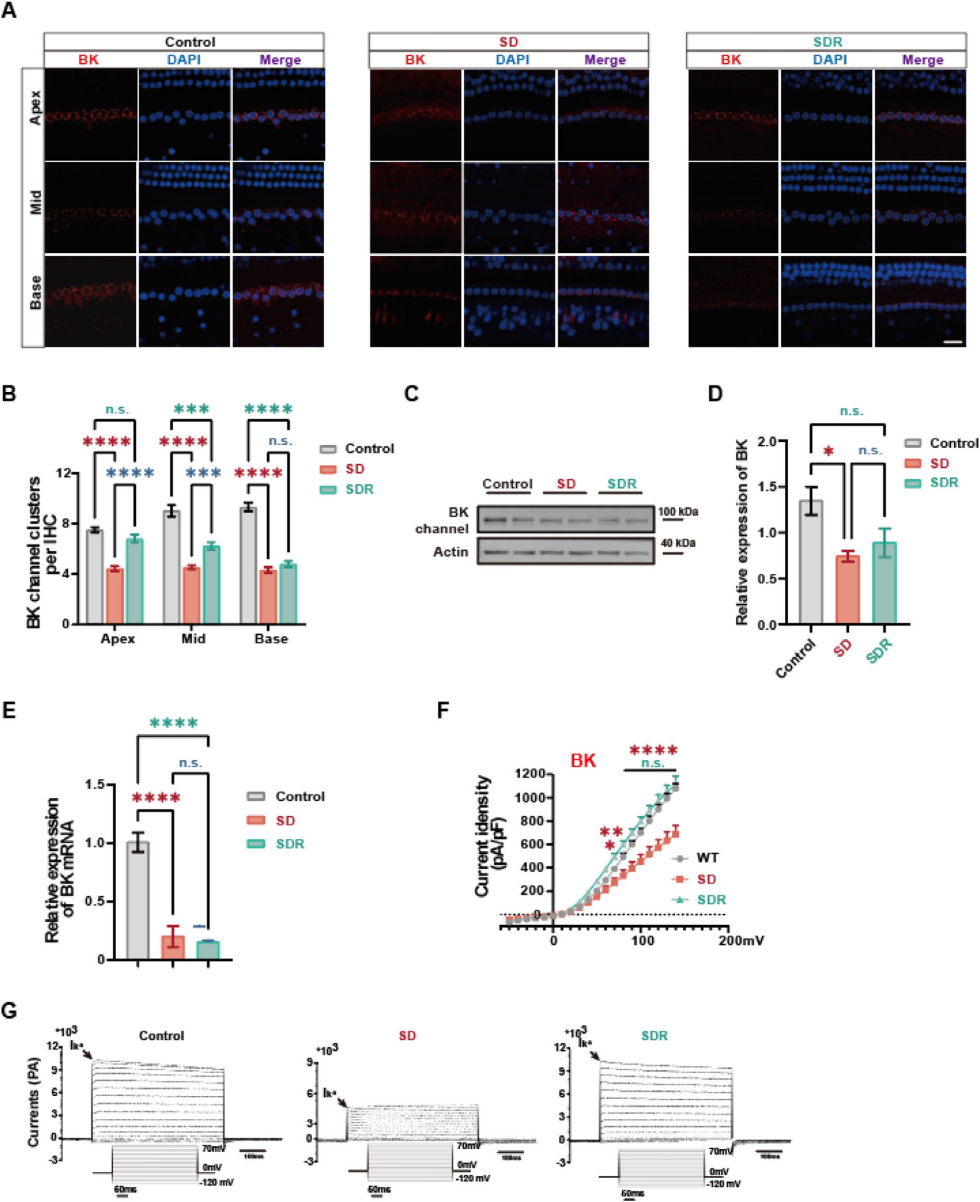
Persistent Inflammation Drives Irreversible, Tonotopic Suppression of BK Channels. (A) Representative confocal images of the OC showing BK channel immunofluorescence (red) at the neck of IHC, with nuclei counterstained with DAPI (blue). Scale bar, 50 µm. (B) Quantification of the number of BK channel immune‐positive clusters per IHC across the three cochlear turns. (C) Representative western blot of whole cochlear lysates probed for BK channel protein (∼100 kDa) and β‐actin as a loading control. (D) Quantification of BK channel protein levels relative to β‐actin. (E) RT‐qPCR analysis of Kcnma1 (BK channel) mRNA levels in whole cochleae. (F) Current‐voltage (*I–V*) relationship plots for the isolated large‐conductance (BK) current density in the cochlear apex. (G) Representative whole‐cell patch‐clamp recordings from the neck of IHC in the cochlear apex. Data are presented as mean ± SEM. Statistical significance was determined by two‐way ANOVA (B) or one‐way ANOVA (D, E) with Tukey's multiple comparisons test (*n.s*., not significant; ^*^ *p* < 0.05, ^***^ *p* < 0.001, ^****^ *p* < 0.0001). n = 6 mice per group.

We first validated this at the protein level using immunofluorescence. In control animals, BK channel protein formed distinct clusters along the neck of IHCs (Figure 6A). Following SD, the expression and clustering of BK channels were significantly reduced across all cochlear turns. Strikingly, in the SDR group, BK expression recovered in the low‐frequency apex but remained profoundly suppressed in the mid‐ and high‐frequency middle and basal turns (Figure 6A,B). This irreversible, tonotopically‐specific loss of a critical ion channel provides a compelling molecular explanation for the tonotopic pattern of the persistent hearing loss.

Western blot and RT‐qPCR analyses of whole cochlear lysates corroborated these findings at a global tissue level. Total BK protein levels were significantly reduced in the SD group and failed to fully recover in the SDR group (Figure 6C,D). Correspondingly, *Kcnma1* mRNA was markedly downregulated in the SD group and remained significantly suppressed after the recovery period (Figure 6E). This confirms that the persistent inflammatory state within the cochlea leads to a lasting transcriptional repression of this critical ion channel, with the most severe impact in the cochlear regions responsible for high‐frequency hearing.

To directly link this molecular suppression to a functional deficit, we performed whole‐cell patch‐clamp recordings on IHCs from the cochlear apex, the region where we observed molecular recovery. Using a voltage‐clamp protocol to isolate potassium currents (Figure S5A), we found that the fast‐activating outward current (Ikf), which is largely carried by BK channels, was significantly attenuated in IHCs from the SD group compared to controls (Figure 6F,G). A similar pattern was observed for the slow‐activating potassium current (Iks), which was also significantly diminished in the SD group (Figure S5B). Consistent with the molecular data, both Ikf and Iks currents in IHCs from SDR animals were fully restored to control levels (Figure 6F,G). To ensure these current changes were not artifacts of general cellular decline, we measured key indicators of cell health. Both membrane capacitance (ΔCm) and resting membrane potential (RMP) remained stable across all three groups, confirming preserved cellular integrity (Figure S5C,D).

This direct evidence demonstrates that the molecular recovery of BK channel expression in the cochlear apex translates to a complete restoration of its physiological function. Conversely, the irreversible high‐frequency hearing loss is the direct functional consequence of the persistent, inflammation‐driven suppression of BK channel expression and function in the cochlear base.

### 2.7 Pharmacological Intervention Targeting the ROS/BK Channel Axis Rescues SD‐Induced Persistent Cochlear Ribbon Synapse Loss

**FIGURE 7:**
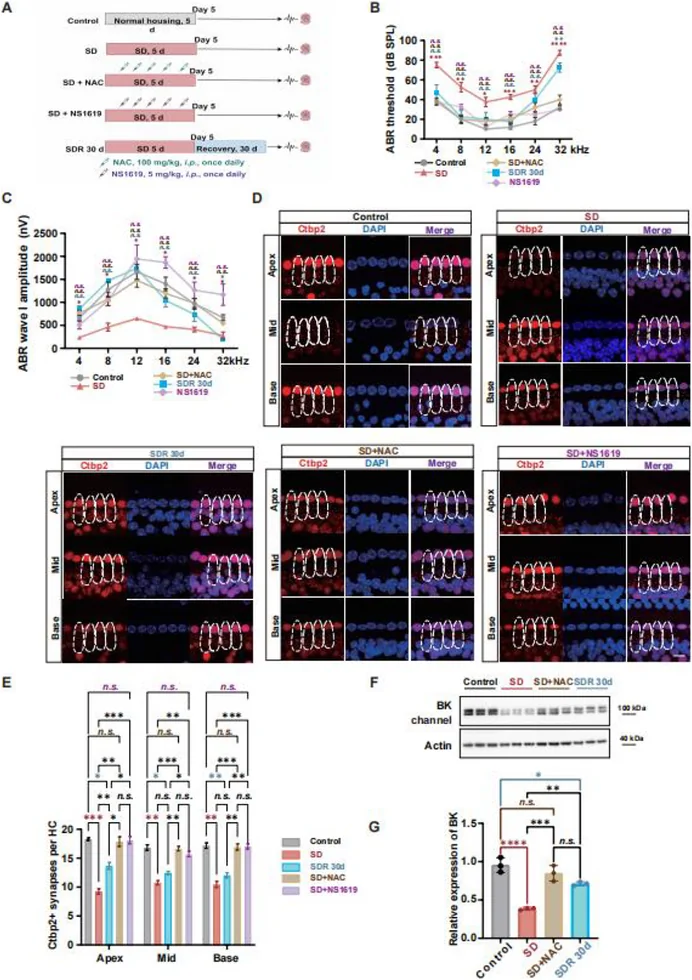
Pharmacological rescue of the oxidative stress–BK channel axis prevents SD‐induced auditory neuropathy. (A) Schematic illustration of the experimental design. Mice underwent normal housing (Control), 5 days of SD, SD with daily *i.p*. administration of NAC (100 mg/kg; SD+NAC), SD with daily *i.p*. administration of the BK‐channel activator NS1619 (5 mg/kg; SD+NS1619), or 5 days of SD followed by 30 days of recovery (SDR 30d). NAC and NS1619 were administered once daily throughout the 5‐day SD period. ABR measurements and cochlear collection were performed at the indicated endpoints. (B, C) ABR thresholds (B) and ABR wave I amplitudes (C) at frequencies ranging from 4 to 32 kHz. Colored annotations above individual data points indicate comparisons between the corresponding treatment group and the Control group at each frequency. (D) Representative confocal images of CtBP2+ presynaptic ribbons in the apical, middle, and basal turns of the cochlea. CtBP2 is shown in red and nuclei are counterstained with DAPI (blue). IHCs are outlined by white dashed lines. Scale bar, 10 µm. (E) Quantification of CtBP2+ ribbon puncta per IHC in the apical, middle, and basal cochlear turns. (F, G) Representative western blots (F) and densitometric quantification (G) of BK‐channel protein expression in whole‐cochlear from the Control, SD, SD+NAC, and SDR 30d groups. (H, I) Representative western blots (H) and densitometric quantification (I) of BK‐channel protein expression in whole‐cochlear from the Control, SD, SD+NS1619, and SDR 30d groups. (J) Representative confocal images showing BK‐channel immunoreactivity in inner hair cells from the apical, middle, and basal cochlear turns. BK‐channel staining is shown in red and nuclei are counterstained with DAPI (blue). Scale bar, 10 µm. (K) Quantification of BK‐channel‐positive clusters per inner hair cell across the three cochlear turns. Statistical significance was determined using two‐way ANOVA followed by Tukey's multiple‐comparisons test for frequency‐ and cochlear‐region‐dependent analyses (B, C, E, and K) and one‐way ANOVA followed by Tukey's multiple‐comparisons test for western blot analyses (G and I). ^*^ *p* < 0.05, ^**^ *p* < 0.01, ^***^ *p* < 0.001, and ^****^ *p* < 0.0001; n.s., not significant.

Having established that a transient oxidative stress wave (Result 2.4) precedes a sustained neuroinflammatory response that culminates in the tonotopically restricted, irreversible suppression of BK channels (Result 2.6), we next sought to establish direct causality within this pathway and to determine whether pharmacological intervention during the acute phase of SD could prevent the ensuing persistent auditory pathology (Figure 7A).

We first asked whether the persistent high‐frequency deficit observed after 14 days of recovery reflected a genuinely irreversible lesion, or merely an incomplete and delayed recovery process. To this end, we extended the recovery period to 30 days (SDR 30d). Mice in the SDR 30d group continued to exhibit significantly elevated ABR thresholds selectively at high frequencies (24 and 32 kHz; Figure 7B), a persistently reduced wave I amplitude (Figure 7C), Ctbp2+ synapse loss restricted to the cochlear base (Figure 7D,E), and sustained suppression of BK channel protein and clustering in the middle and basal turns (Figure 7F–K), essentially replicating the tonotopic damage pattern observed at 14 days. These findings confirm that the high‐frequency cochlear ribbon synapse loss and BK channel suppression represent a genuinely persistent pathological end‐state rather than a slowly resolving one.

We next tested whether this persistent phenotype could be averted by intervening during the initial, oxidative stress‐dominated phase of injury. Systemic administration of the antioxidant N‐acetylcysteine (NAC) throughout the 5‐day SD protocol (SD+NAC) robustly prevented the development of persistent pathology. ABR thresholds in the SD+NAC group were statistically indistinguishable from controls across the entire tested frequency range, including the high frequencies that remained persistently elevated in the SD and SDR 30d groups (Figure 7B). This functional protection was paralleled by preserved wave I amplitudes (Figure 7C) and, critically, by near‐complete preservation of Ctbp2+ ribbon synapse density in the basal turn (Figure 7D,E). This is the very cochlear region in which ribbon synapse loss was otherwise irreversible. NAC co‐treatment also prevented the loss of BK channel protein, as confirmed by western blot (Figure 7F,G) and by immunofluorescence quantification of BK clusters across all three cochlear turns (Figure 7J,K). These results demonstrate that neutralizing the initial oxidative insult is sufficient to interrupt the downstream cascade linking SD to chronic neuroinflammation, BK channel suppression, and persisting cochlear ribbon synapse loss, thereby establishing oxidative stress as a causally necessary initiating event in this pathway.

To determine whether BK channel suppression is not merely a downstream biomarker but a functionally required mediator of the persistent phenotype, we asked whether direct pharmacological restoration of BK channel activity during SD, independent of any antioxidant action, would similarly confer protection. Co‐administration of the BK channel opener NS1619 during the SD protocol (SD+NS1619) fully prevented the emergence of high‐frequency threshold elevation and wave I amplitude reduction (Figure 7B,C), and, notably, also preserved Ctbp2+ synapse density in the basal turn to a degree comparable with NAC treatment (Figure 7D,E). Western blot and immunofluorescence analyses further confirmed that NS1619 preserved both total BK channel protein levels (Figure 7H,I) and its characteristic clustering pattern along the neck of IHCs across all cochlear turns, including the mid and basal regions that otherwise remained suppressed in the SDR 30d group (Figure 7J,K). Because NS1619 acts as a direct allosteric activator of BK channel gating rather than as a general antioxidant, its protective efficacy against cochlear ribbon synapse loss indicates that loss of BK channel‐mediated hyperpolarization, rather than oxidative damage per se, constitutes a proximal and mechanistically necessary step linking the inflammatory cascade to synaptic degeneration, plausibly by permitting excessive depolarization‐driven Ca^2+^ influx and glutamate excitotoxicity at the IHC‐SGN synapse in its absence.

Collectively, these pharmacological rescue experiments delineate a linear and experimentally tractable pathogenic sequence—oxidative stress, sustained neuroinflammation, and BK channel suppression—that culminates in persistent, tonotopically restricted cochlear ribbon synapse loss and high‐frequency hearing loss. Importantly, interventions targeting either the most upstream trigger (oxidative stress, via NAC) or the critical downstream effector (BK channel function, via NS1619) were each independently sufficient to prevent this persistent pathology, identifying two mechanistically distinct and potentially actionable therapeutic strategies for preventing SD‐induced irreversible auditory neuropathy.

## 3 Discussion

Our study reveals that SD drives lasting auditory damage through a pathogenic switch from a transient systemic insult to a self‐sustaining neuroinflammatory state within the cochlea. This work redefines the pathophysiology of stress‐induced hearing loss, demonstrating that it is not a passive consequence of systemic fatigue but an active disease process driven by a local “neuroinflammatory memory”. Crucially, by integrating targeted pharmacological interventions (NAC and NS1619) and rigorous behavioral controls, we have moved beyond correlation to establish a definitive causal axis linking systemic sleep loss, ROS‐driven inflammation, and specific ion channel suppression. This mechanistic insight provides a biological basis for the strong clinical association we and others have observed between sleep disorders and SNHL, offering a new framework for understanding how a common systemic stressor can precipitate a chronic, debilitating sensory neuropathy.

A fundamental challenge in neuroscience is to understand the mechanisms that convert transient, systemic physiological insults into chronic, localized neural circuit dysfunction. The nervous system is constantly exposed to systemic stressors [13, 14], yet the process by which an acute, body‐wide event like SD can precipitate a persistent, circuit‐specific pathology remains poorly defined. This temporal and spatial disconnect implies the existence of local, self‐perpetuating pathological mechanisms that effectively store a “memory” of the initial stressor, driving neurodegeneration long after systemic homeostasis has been restored. In this study, by dissecting the multi‐stage cascade from systemic stress to cellular dysfunction, we establish the cochlea as a powerful model for this process and provide a new mechanistic framework for understanding how systemic stress translates into chronic peripheral neuropathy.

Our investigation began by precisely defining the long‐term functional and structural sequelae of acute SD. Consistent with prior work in rats [11], we observed a profound, pan‐frequency elevation in ABR thresholds immediately following a 5‐day SD protocol. However, the critical insight emerged during the recovery phase. Although systemic physiological balance was fully restored after the 14‐ and 30‐day recovery periods, hearing loss persisted specifically at high frequencies (≥24 kHz). The lack of further improvement between day 14 and day 30 confirms that this deficit had reached a stable plateau, rather than being an intermediate stage of a prolonged recovery. This finding is not only a significant refinement of previous preclinical work but also holds profound clinical relevance, as high‐frequency hearing is often the most vulnerable and difficult to restore in patients with SSNHL [15, 16]. The persistence of this deficit, especially the reduced ABR wave I amplitude—a robust proxy for the synchronous firing of the auditory nerve [17, 18]—strongly implicated cochlear synaptopathy (here defined, consistent with its established usage in the field, as the combined loss of presynaptic ribbon synapses and reduced afferent neural output occurring in the absence of significant hair cell loss) as the underlying lesion. This aligns with clinical observations in related conditions like obstructive sleep apnea (OSA), where chronic intermittent hypoxia is thought to cause similar cochlear neurodegeneration [19, 20]. Our morphological analysis provided direct structural evidence for this hypothesis, revealing a significant, and persistent loss of IHC ribbon synapses that precisely mirrored the functional deficit: substantially, though not completely, reversible in the low‐frequency apex but persistent in the high‐frequency basal turn. Notably, this apical recovery was evident at the functional and synaptic level but was not fully mirrored structurally: OHC stereocilia morphology remained incompletely normalized in the apical and middle turns even after functional and synaptic measures had substantially recovered, indicating that distinct cochlear cell types follow dissociable, structure‐specific recovery trajectories following SD.

A critical conceptual hurdle in stress biology is distinguishing the specific effects of sleep loss from generalized systemic stress. To address this, we employed a restraint stress (RS) paradigm. Strikingly, while RS induced comparable systemic stress responses, it failed to elicit the permanent high‐frequency hearing loss and cochlear synaptopathy observed following SD. This pivotal negative control unequivocally demonstrates that the auditory neuropathy we describe is not a generic consequence of HPA axis activation or nonspecific stress, but is uniquely attributable to the specific physiological disruptions caused by SD. We also acknowledge that our 22‐h/day SD paradigm represents a severe, acute stressor rather than the more chronic, partial sleep restriction typically experienced by patients with sleep disorders; this paradigm was selected to reliably and rapidly establish the underlying molecular cascade within a tractable experimental timeframe, and our parallel human cohort data which demonstrates a graded association between habitual sleep disturbance severity and hearing loss, provide independent support for the translational relevance of the pathway identified here. Whether more moderate, chronic sleep restriction produces a qualitatively similar, if quantitatively attenuated, cochlear pathology remains an important question for future investigation.

Furthermore, our findings reveal a remarkable convergence in the downstream pathology between SD‐induced hearing loss and the canonical model of noise‐induced hearing loss (NIHL), yet they diverge critically in their upstream etiology and core pathogenic mechanism. Both pathologies manifest as a high‐frequency SNHL characterized by cochlear synaptopathy [21, 22, 23], oxidative stress [24, 25, 26], and BK channel dysfunction [27]. However, whereas NIHL is precipitated by a direct, localized acoustic trauma leading to acute excitotoxicity and massive metabolic overload [28, 29], the cochlear injury in our model originates from a systemic, neuroendocrine stressor. The most significant distinction lies in the subsequent pathogenic cascade. In NIHL, cochlear damage is largely a direct, dose‐dependent consequence of the acute insult [30, 31]. In contrast, our data reveal a novel two‐stage process for SD: a transient systemic stressor (HPA activation and oxidative stress) ignites a chronic, self‐sustaining local neuroinflammatory program that becomes the primary driver of persistent pathology, long after the initial insult has resolved. This inflammation‐driven synaptopathy represents a more subtle and insidious injury than the overt hair cell death often seen in traumatic NIHL [32, 33] and establishes a new paradigm for stress‐induced cochlear damage.

The uncoupling of the transient trigger from the chronic pathology is the central conceptual advance of this study. The severe cochlear oxidative stress we observed—a direct consequence of HPA axis hyperactivation—resolved completely with recovery, yet the auditory deficits and synaptopathy persisted. This temporal disconnect proves that oxidative stress, while a critical initiator, is not the ultimate driver of the persistent damage. This was definitively proven by our pharmacological intervention: early administration of the ROS scavenger NAC not only blunted the initial oxidative burst but successfully prevented the establishment of the chronic inflammatory state and the subsequent permanent synaptopathy. Our transcriptomic analysis provides the mechanistic explanation for this persistence, revealing a pathological switch within the cochlea's cellular programming. The transcriptome of the SDR group was not one of recovery but of chronic, smouldering inflammation. The sustained upregulation of pro‐inflammatory cytokines (e.g., *Il1b*), chemokines (*Cxcl3*), and complement factors (*Cfb*) points to the establishment of a self‐perpetuating local inflammatory state. We propose that this “neuroinflammatory memory” is the key driver of the lasting synaptopathy and the ongoing transcriptional repression of critical homeostatic genes. This model posits that resident cochlear immune cells, likely microglia‐like cells and macrophages [34, 35], become primed by the initial oxidative insult and transition into a persistently reactive state, creating a toxic microenvironment that prevents synaptic repair and actively promotes neurodegeneration. We note, however, that our transcriptomic dataset was generated from bulk cochlear tissue using a modest number of biological replicates, which limits both the statistical power to detect smaller‐magnitude transcriptional changes and our ability to resolve which specific resident cell population(s) drive this inflammatory signature. Although our targeted protein‐level validation—for example, immunolocalization of BK channel loss specifically to hair cells—provides some cell‐type‐specific corroboration, definitively identifying the cellular origin of this “memory,” and determining whether it is sustained through ongoing paracrine signaling or through stable chromatin remodeling at inflammatory gene loci, will require single‐cell RNA sequencing or sorted‐cell qPCR validation together with ATAC‐seq or ChIP‐seq profiling of key inflammatory loci. We identify these approaches as important priorities for future work.

This sustained inflammatory state directly targeted the expression and function of the BK channel, a crucial regulator of hair cell excitability and cochlear function [36, 37, 38]. First discovered in *Drosophila* [39], BK channels are known to be critical for multiple aspects of mammalian auditory function, including cochlear development [40], afferent synaptic transmission [41], and providing essential protection against acoustic trauma and aging [38, 41, 42]. Crucially, prior research has shown that reduced BK expression can lead to abnormal ABR wave I amplitudes [38], directly linking this channel to our primary functional finding. Our data solidify this connection in the context of SD. The transcriptional repression of *Kcnma1* manifested as a striking tonotopic gradient of protein loss: expression was restored in the low‐frequency apex but remained suppressed in the mid‐to‐basal turns. This elegant result resolves the apparent discrepancy between whole‐cochlea assays (which showed incomplete recovery) and our electrophysiological data, which confirmed a specific and complete restoration of BK‐mediated currents in apical IHCs. Most importantly, direct pharmacological activation of BK channels using NS1619 successfully rescued the SD‐induced permanent hearing loss and synaptic damage. Together with the NAC rescue described above, which blocks the initiating oxidative trigger, this independent restoration of BK channel function downstream establishes a causal chain running from oxidative stress through chronic inflammation to BK channel dysfunction and synaptopathy, as each of these two mechanistically non‐overlapping interventions was independently sufficient to prevent the pathological outcome. This establishes BK channel suppression not merely as a correlative marker, but as the indispensable molecular effector of the inflammation‐driven auditory decline. Thus, functional recovery of hearing in the apex is driven by the resolution of inflammation and subsequent restoration of BK channels, while persistent high‐frequency hearing loss is explained by the persistent, inflammation‐driven absence of these critical channels in the cochlear base. This raises the question of why the basal turn is disproportionately susceptible to this sustained molecular suppression despite a comparable initial oxidative insult across cochlear regions. We suggest that oxidative stress functions as a common initiating trigger that interacts with a pre‐existing, intrinsic basal‐to‐apical gradient of vulnerability, rather than acting as the sole determinant of the frequency‐specific outcome. Basalhair cells and their surrounding structures are known to exhibit higher metabolic demand and comparatively lower baseline antioxidant reserve than apical structures [43], properties that could plausibly lower the threshold at which a transient oxidative insult escalates into a self‐sustaining local inflammatory response specifically in the base. In addition, SD‐associated sympathetic overactivation may disproportionately compromise microvascular perfusion of the basal turn [44, 45], further impairing local antioxidant defense and clearance of inflammatory mediators in this region. We were unable to directly test these possibilities in the present study, and we identify region‐resolved comparison of antioxidant capacity, metabolic demand, microvascular perfusion, and immune cell density between the apical and basal turns, both at baseline and following SD, as an important direction for future work to mechanistically explain this tonotopic divergence.

Our findings position SD‐induced hearing loss within an emerging paradigm of systemic synaptic vulnerability that extends far beyond the central nervous system. It is well‐established that SD activates the HPA axis and induces neuroinflammation within the brain, contributing to cognitive deficits [3, 46] and mood disorders [47, 48]. Our study demonstrates that the cochlea is not an isolated target but rather a sentinel of this systemic stress. This central stress state is driven by the dysregulation of key sleep‐wake circuits; for example, the VTA contains distinct populations of GABAergic neurons that promote sleep and glutamatergic neurons that drive wakefulness, the imbalance of which is a core feature of SD [49]. Concurrently, the failure to engage key sleep‐promoting neurons, such as the galanin‐expressing neurons of the preoptic area that are fundamental for homeostatic sleep pressure, represents a critical breakdown that perpetuates the stressful, wakeful state [50]. Indeed, there are strong pathophysiological parallels in other peripheral systems where synaptic integrity is crucial. For instance, SD is increasingly linked to enteric nervous system dysfunction, leading to gut motility disorders and visceral hypersensitivity, pathologies underpinned by synaptic dysregulation within the gut wall [51, 52]. Similarly, autonomic neuropathy may contribute to the cardiovascular instability, such as hypertension [53] and arrhythmias [54], frequently observed after sleep loss. In this context, the cochlear synaptopathy we describe is likely not a unique phenomenon but rather one manifestation of a systemic, inflammation‐driven assault on sensitive neural circuits. The cochlea, with its exquisite sensitivity, tonotopic organization, and accessibility for functional testing, thus serves as a critical window into this widespread pathology.

Several limitations of the present study merit explicit acknowledgment. First, although co‐localization of presynaptic ribbons with postsynaptic densities is considered the morphological gold standard for defining an intact afferent synapse, our attempts to immunolabel postsynaptic markers (GluA2, PSD95) in adult cochlear whole‐mounts were unsuccessful despite extensive optimization, a technical limitation also reported by other groups working with adult, decalcified cochlear tissue. We therefore rely on the tight spatial and temporal correspondence between presynaptic ribbon loss (CtBP2) and reduced ABR wave I amplitude—a well‐established functional proxy for synchronized auditory nerve firing [55, 56]—as our primary evidence of impaired synaptic transmission. This evidence, however, remains indirect with respect to transmission and plasticity at the level of individual synapses, and direct confirmation using transmission electron microscopy or single‐synapse electrophysiological recording remains an important goal for future work. Second, our clinical cohort did not assess tinnitus, which is a well‐recognized independent contributor to impaired sleep quality [57]; we cannot exclude the possibility that tinnitus mediates or contributes to part of the association we observed between hearing loss severity and sleep disturbance, and future prospective studies should incorporate validated tinnitus instruments (e.g., the Tinnitus Handicap Inventory or Tinnitus Functional Index) alongside longitudinal sleep assessments. Correspondingly, our animal model did not include behavioral assays of tinnitus‐like perception (e.g., gap‐detection acoustic startle testing), and we therefore cannot exclude a contribution of tinnitus to the phenotypes described here. Finally, the present study was designed to test the forward direction of the sleep‐hearing relationship, namely that sleep loss drives cochlear pathology; whether cochlear synaptopathy and hearing loss can, in turn, secondarily impair sleep quality or cognitive function—suggesting a self‐reinforcing bidirectional loop consistent with what is observed clinically—remains an open question that will require direct assessment of sleep architecture, for example by EEG/EMG polysomnography, following isolated peripheral auditory injury in future animal studies.

In conclusion, our study provides compelling clinical and experimental evidence that SD acts as an active driver of SNHL rather than a mere comorbidity. Mechanistically, we reveal that SD triggers a pathogenic switch from transient systemic stress to localized, self‐sustaining cochlear neuroinflammation. This ROS‐driven cascade suppresses BK channel activity and induces synaptic ribbon loss, culminating in persistent high‐frequency auditory deficits. These findings fundamentally redefine the pathophysiology of stress‐associated hearing impairment, shifting the paradigm from a passive consequence of systemic fatigue to an active, targetable disease process. While future longitudinal studies are warranted to fully unravel the bidirectional interplay between auditory dysfunction and sleep architecture, our current work establishes the ROS‐neuroinflammation‐BK channel axis as a promising therapeutic target for mitigating sleep‐disorder‐related hearing loss.

## 4 Materials and Methods

### 4.1 Human Subjects and Ethical Approval

Patients with SSNHL were prospectively recruited from the Department of Otolaryngology at the Third Affiliated Hospital of Sun Yat‐sen University between September 1, 2023, and December 31, 2024. Diagnosis and inclusion were based on criteria established by the Clinical Practice Guideline for Sudden Hearing Loss. To exclude confounding pathologies, all patients underwent a comprehensive neuroimaging protocol, including magnetic resonance imaging (MRI) of the internal auditory canal, computed tomography (CT) of the temporal bone, and magnetic resonance angiography (MRA) of the brain. This screening definitively ruled out conditions such as acoustic neuroma, meningioma, inner ear malformations, and stroke. A control group of healthy individuals with no history of hearing disorders was concurrently recruited from the hospital's physical examination center. Hearing thresholds for all participants were determined by standard pure‐tone audiometry. This study was conducted in full accordance with the principles outlined in the Declaration of Helsinki. The study protocol received formal approval from the Ethics Committee of the Third Affiliated Hospital of Sun Yat‐sen University. Written informed consent was obtained from every participant prior to their enrollment. The consent process ensured all individuals understood that their participation involved both hearing function and sleep quality assessments, and that their anonymized data would be used for research purposes.

### 4.2 Assessment of Pure Tone Average (Pta) and Sleep Quality and Data Analysis

Within 24 h of hospital admission, all participants underwent a standardized assessment in a quiet clinical setting. Demographic data (age, gender) were recorded. Auditory function was evaluated by pure‐tone audiometry using a calibrated audiometer (Tingmei 1081, Dandelion) within a sound‐attenuating booth compliant with ISO 8253 standards. The PTA was calculated as the mean air‐conduction threshold across 500, 1000, 2000, and 4000 Hz. Subjective sleep quality was assessed using the PSQI and a 10‐point VAS for sleep (0 = best, 10 = worst), with all questionnaires administered under the supervision of trained staff. All statistical analyses were performed using SPSS (Version 27.0). After assessing for normality with the Shapiro–Wilk test, between‐group comparisons for non‐normally distributed continuous variables (PTA, PSQI, VAS) were conducted using the Mann‐Whitney *U* test, while the Chi‐square test was used for categorical data (gender). Spearman's rank correlation was employed to analyze the relationships between PTA and sleep quality metrics. A two‐tailed *p*‐value < 0.05 was considered statistically significant.

### 4.3 Animals and Ethical Approval

Male C57BL/6J mice, aged 6–8 weeks and weighing 20 to 24 g, were used in this experiment. All the mice employed in the study were specific pathogen‐free (SPF) grade, carrying no specific pathogens. The mice were housed at a temperature of 22°C–24°C, and the feeding environment maintained a 12 h light/dark cycle to preserve normal basal rhythms. The mice were randomly assigned to eight groups: Control, SD, sleep deprivation recovery for 7 days (SDR 7 d), sleep deprivation recovery for 14 days (SDR 14 d), sleep deprivation recovery for 30 days (SDR 30 d), sleep deprivation with NAC treatment, sleep deprivation with NS1619 treatment and restraint stress treatment. The experimental mice were supplied by the animal house of the State Key Laboratory of Bioelectronics at Southeast University. The feeding and experimental procedures for the mice were approved by the Southeast University Animal Care and Use Committee (experiment number: 20221128003), which were aligned with the National Institutes of Health Guidelines for the Care and Use of Laboratory Animals. We did our best to alleviate animal suffering and to decrease the quantity of animals used.

### 4.4 Animal SD Procedures

In this experiment, an SD apparatus (25 cm in diameter and 30 cm in height) (Shanghai Xinruan Information Technology Co., Ltd., #XR‐XS108) was employed for SD modelling (Figure S1A). Mice in the experimental groups (SD, SDR 7d, SDR 14d, SDR 30d) were placed into the SD apparatus in batches. Given the limited size of the device, the maximum number of mice placed into the SD apparatus at one time was set to 10 to prevent overcrowding in the experimental environment, which might otherwise deteriorate the modelling effect. The system supplies food and water. To guarantee the validity of the experimental data, the control mice were housed in the same environment. To induce SD in mice, intermittent SD was carried out for five consecutive days. The SD period was from 21:00 to 19:00 of the next day, lasting for 22 h, and the sleep rest period was from 19:00 to 21:00, lasting for 2 h (Figure 2A). To guarantee the effectiveness of the modelling, the bedding in the apparatus could be changed during the experiment to prevent the mice from falling asleep due to environmental adaptation.

### 4.5 Auditory Brainstem Response (ABR)

1% (w/v) pentobarbital sodium solution was configured, and mice were anesthetized at a dose of 70 mg/kg of body weight. ABR waveforms were measured using the TDT (Tucker‐Davis, #system III). Electrodes were placed on the mice: a recording electrode (red) was placed subcutaneously in the midline of the brain, a reference electrode (green) was placed subcutaneously behind the ear in the test ear, and a grounding electrode (black) was placed subcutaneously in the hindlimb or behind the ear in the contralateral ear. The mice were positioned with their backs upward to ensure that the distance from the ear to the speaker was about 7 cm. Pure tone detection: short pure tones of 4, 8, 12, 16, 24, and 32 kHz were used as the acoustic stimuli, and the acoustic evoked potentials were averaged over 1024 times with a scan time of 10 ms. The intensity of acoustic stimulation at each frequency was started from 90 dB and was decreased in 10 dB steps according to the experimental needs until no repetitive ABR waveforms were detected. Waveforms were exported via Biosig software, hearing thresholds were recorded, and data were analyzed.

### 4.6 ABR Wave I Amplitude Statistics

The vertical distance from the negative peak before the start of wave I to the positive peak of wave I was determined from the derived ABR waveform curve. As shown in Figure 2C, the horizontal coordinate represents the wave latency of ABR and the vertical coordinate represents the ABR amplitude magnitude. The red line is the ABR wave I amplitude value. The wave latency of wave I in normal mice is 0.9–1.4 ms and wave I can be determined accordingly.

### 4.7 Immunofluorescent Staining

Cochleae were fixed in 4% (w/v) paraformaldehyde (PFA) for 24 h at 4°C temperature and then washed with phosphate buffer saline (PBS). Cochleae were decalcified with 0.5 M EDTA for 12–24 h at room temperature. The cochlear basilar membrane (BM) tissues were dissected in cold PBS, which were divided into three equal turns (apical, middle, and basal turns). And then blocked with blocking solution (5% donkey serum, 0.5% Triton X‐100, 0.02% sodium azide, and 1% bovine serum albumin in pH7.4 PBS) for 1 h at room temperature. The cochleae were then incubated with primary antibodies diluted in phosphate‐buffered saline with Triton (PBT)‐1 (2.5% donkey serum, 0.1% Triton X‐100, 0.02% sodium azide, and 1% bovine serum albumin in pH7.4 PBS) at 4°C overnight. After washing with 0.5% PBST (0.1% Triton X‐100 in pH7.4 PBS) for three times (5min each time), the cochleae were incubated with secondary antibody, diluted 1:400 in PBT‐2 (0.1% Triton X‐100 and 1% bovine serum albumin in pH7.4 PBS), for 1h at room temperature. After another three times of washing with 0.1% PBST (5min each time), the cochleae were mounted in antifade fluorescence mounting medium (DAKO, #S3023). The primary antibodies used in this study were anti‐myosin7a (Proteus Bioscience, 25–6790), anti‐tuj1 (Abcam, Ab78078, 1:400), anti‐ctbp2 (BD Biosciences 612044, 1:400), anti‐BK (Alomone Labs, APC‐021, 1:200), anti‐DHE (Beyotime, S0063, 1:200), anti‐MDA (Abcam, ab243066, 1:1000). The secondary antibodies are donkey anti‐rabbit (Invitrogen, A‐31572, 1:400), donkey anti‐mouse (Invitrogen, A‐21202, 1:400), donkey anti‐mouse IgG1 (Invitrogen, A‐21202, 1:400) and DAPI (Solarbio, C0060, 1:1000 dilution). A Zeiss LSM 880/900 confocal microscope was used to obtain the fluorescence images and all images were analyzed by Image J software.

### 4.8 RNA Extraction and Real‐Time Quantitative PCR (RT‐qPCR)

Cochleae were used to extract total RNA with Trizol (Vazyme, R411‐01). RNA was reverse transcribed into cDNA by using Reverse Transcription Kit (Vazyme, R333). The reverse transcription PCR conditions were 42°C for 60 min followed by 70°C for 5 min. Then the Taq Pro‐Universal SYBR qPCR Master Mix Kit (Vazyme, Q712) was used to perform the RT‐qPCR to quantify the gene expression levels with β‐actin as the reference endogenous gene. The primers were as follows:

β‐actin: (F) 5’‐ ACG GCC AGG TCA TCA CTA TTG‐3’; (R) 5’‐AGG GGC CGG ACT CAT CGT A‐3’.

Cav1.3: (F)5’‐TTT GAC AAT GTC CTT TCG GCT‐3’; (R)5’‐TTC TCA CCG TTT GAA TCA ATA GCT‐3’.

BK: (F)5’‐TGG TGC CCT CGT AAT ATA CTT CA‐3’; (R)5’‐ CAG CTT ATC GTT GGC TGC AAT ‐3’.

SK: (F)5’‐GCT CAA CCA AGT CCG CTT C‐3’; (R)5’‐ GTG ATC GGA ATC AGC CAC AGT ‐3’.

The RT‐qPCR steps were 60 s at 50°C, 10 min at 95°C followed by 40 cycles of 15 s at 95°C with 1min at 60°C, then 5 s at 95°C, 5 s at 65°C, and 5 s at 95°C. Gene expression was quantified using the ∆∆CT method as follows: ∆CT experimental group = CT experimental group—CT experimental group's internal reference, ∆CT control group = CT control group—CT control group's internal reference, ∆∆CT = ∆CT experimental group—∆CT control group, and 2^‐∆∆CT values were calculated.

### 4.9 Protein Extraction and Western Blot

Cochleae from mice of different ages were used to extract protein with 200 µL RIPA buffer (Beyotime, S3090) supplemented with 4 µL 50× phosphatase inhibitor cocktail (Roche, #04693132001). The protein samples were added to Omni‐Easy Protein Sample Loading Buffer (Epizyme, LT101), boiled at 98°C for 10 min, and separated by SDS‐PAGE. Then it was transferred to PVDF membrane with pore size 0.45 or 0.22 µm (Millipore, IPVH00010). The membrane was blocked with 5% (v/v) milk mixed with 1 × TBST buffer (1 × Tris‐base saline solution at pH7.4, 0.1% [v/v] Tween‐20) at room temperature for 1 h and incubated with primary antibody overnight. The primary antibody of BK channel was used to detect the expression of BK, and primary antibody of β‐Actin (ABclonal, AC026 1:50000) were serve as reference proteins. After washing with 1 × TBST buffer for three times (10 min each time), the blot membranes were incubated at room temperature for 2 h with the corresponding IgG HRP‐conjugated secondary antibody (ABclonal, AS014 1:5000). After repeated washing for three times by 1 × TBST buffer (10 min each time), the Chemiluminescent Substrate Kit (NCM biotech, p10300B) was used to detect protein signals, and imaging was performed on the Tanon 2500R system (Tanon, China).

### 4.10 RNA Extraction and Library Construction

After deep anesthesia, the mice were decapitated, and their cochleae were carefully extracted, excluding the vestibular tissue. Three mice were used for each age group. Total RNA was extracted and purified utilizing TRIzol reagent (Invitrogen, Carlsbad, CA, USA) in accordance with the manufacturer's guidelines. The quantity and purity of each RNA sample were quantified using NanoDrop ND‐1000 (NanoDrop, Wilmington, DE, USA). The integrity of the RNA was evaluated by Bioanalyzer 2100 (Agilent, CA, USA) with a RIN number exceeding 7.0, and this was corroborated through electrophoresis with denaturing agarose gel. Poly (A) RNA was purified from 1 µg total RNA employing Dynabeads Oligo (dT)25‐61005 (Thermo Fisher, CA, USA) through two rounds of purification. Subsequently, the poly(A) RNA was fragmented into smaller pieces using the Magnesium RNA Fragmentation Module (NEB, cat.e6150, USA) at 94°C for 5–7 min. The cleaved RNA fragments were then reverse‐transcribed to generate cDNA using SuperScript II Reverse Transcriptase (Invitrogen, cat. 1896649, USA), which was subsequently utilized to synthesize U‐labeled second‐stranded DNAs with *E. coli* DNA polymerase I (NEB, cat.m0209, USA), RNase H (NEB, cat.m0297, USA), and dUTP Solution (Thermo Fisher, cat.R0133, USA). An A‐base was added to the blunt ends of each strand, preparing them for ligation to the indexed adapters. Each adapter features a T‐base overhang to facilitate the ligation to the A‐tailed fragmented DNA. Single‐ or dual‐index adapters were ligated to the fragments, and size selection was conducted using AMPureXP beads. Following treatment with the heat‐labile UDG enzyme (NEB, cat.m0280, USA) on the U‐labeled second‐stranded DNAs, the ligated products were amplified via PCR under the following conditions: initial denaturation at 95°C for 3 min; 8 cycles of denaturation at 98°C for 15 s, annealing at 60°C for 15 s, and extension at 72°C for 30 s; followed by a final extension at 72°C for 5 min. The average insert size for the final cDNA library was 300 ± 50 bp. Finally, we conducted 2 × 150 bp paired‐end sequencing (PE150) on an Illumina Novaseq 6000 (LC‐Bio Technology CO., Ltd., Hangzhou, China) adhering to the vendor's recommended protocol.

### 4.11 Bioinformatics Analysis of RNA‐Seq

Fastp software (https://github.com/OpenGene/fastp) were used to remove the reads that contained adaptor contamination, low quality bases and undetermined bases with default parameter. Then sequence quality was also verified using fastp. We used HISAT2 (https://ccb.jhu.edu/software/hisat2) to map reads to the reference genome of Mus musculus GRCm39. The mapped reads of each sample were assembled using StringTie (https://ccb.jhu.edu/software/stringtie) with default parameters. Then, all transcriptomes from all samples were merged to reconstruct a comprehensive transcriptome using gffcompare (https://github.com/gpertea/gffcompare/). After the final transcriptome was generated, StringTie and was used to estimate the expression levels of all transcripts. StringTie was used to perform expression level for mRNAs by calculating FPKM (FPKM = [total_exon_fragments/mapped_reads(millions) × exon_length(kB)]). The differentially expressed mRNAs were selected with fold change > 2 or fold change < 0.5 and with parametric F‐test comparing nested linear models (*p* value < 0.05) by R package edgeR (https://bioconductor.org/packages/release/bioc/html/edgeR.html). DAVID software (https://david.ncifcrf.gov/) facilitated KEGG enrichment analysis and GO enrichment analysis for differentially expressed genes.

### 4.12 Electrophysiological Recordings

Electrophysiological recordings were performed on inner hair cells (IHCs) acutely isolated from the apical turn of the cochlea. Mice (postnatal 6–8 weeks, male) from the Control, SD, and SDR 14 d groups were deeply anesthetized with pentobarbital sodium (70 mg/kg) and decapitated. The temporal bones were rapidly removed and placed in ice‐cold, oxygenated (95% O_2_/5% CO_2_) HEPES‐buffered artificial cerebrospinal fluid (ACSF) containing (in mM): 115 NaCl, 11.2 NaHCO_3_, 6 KCl, 1.3 NaH_2_PO_4_, 1.3 MgCl_2_, 1.3 CaCl_2_, and 11 D‐glucose (pH 7.4, 310 mOsm). Under a dissecting microscope, the cochlear capsule was carefully opened, and the organ of Corti was micro‐dissected from the apical turn. The isolated tissue was then transferred to a recording chamber mounted on an inverted microscope (Olympus BX51WI) and continuously perfused with normal ACSF at a flow rate of 1–2 mL/min at room temperature (22°C–24°C).

IHCs were visually identified using a 60 × water‐immersion objective with differential interference contrast (DIC) optics, based on their characteristic cylindrical morphology, the presence of distinct stereocilia bundles in V‐shape, and their specific position within the sensory epithelium. Whole‐cell patch‐clamp recordings were performed using an Axon 7500B amplifier (Molecular Devices). Patch pipettes were pulled from borosilicate glass capillaries to a resistance of 10–15 MΩ when filled with the internal solution. The internal solution contained (in mM): 135 KCl, 5 HEPES‐KOH, 2.5 Na_2_ATP, 3.5 MgCl_2_, 5 EGTA‐KOH, and 0.1 CaCl_2_ (pH 7.35, 325 mOsm).

For voltage‐clamp recordings, IHCs were held at a holding potential of −70 mV. Outward potassium currents were elicited by a series of depolarizing voltage steps from −60 mV to +60 mV in 10 mV increments (duration: 200 ms). To isolate distinct potassium current components, the recorded total outward current was analyzed based on its kinetic properties. Specifically, the steady‐state outward potassium current (Ikf), which activates rapidly and exhibits minimal inactivation during the depolarizing pulse, was identified as the BK channel‐mediated current, consistent with its well‐characterized large‐conductance, fast‐activating phenotype in IHCs. The slowly activating potassium current (Iks), which exhibits a characteristic slow rise time and sustained plateau during the voltage step, was identified as the SK channel‐mediated current, based on its distinct activation kinetics. All currents were low‐pass filtered at 2 kHz and digitized at 10 kHz using a Digidata 1550B digitizer (Molecular Devices). Series resistance was compensated by 70%–80%, and recordings with series resistance changes >20% were excluded from analysis. Current amplitudes were normalized to the cell membrane capacitance (Cm) to calculate current density (pA/pF). Only cells with stable resting membrane potentials (RMP < −50 mV) and stable access resistance were included in the final analysis. Data acquisition and analysis were performed using Clampex 10.7 and Clamfit 10.7 software (Molecular Devices), and final graphical representation was made using GraphPad Prism 9.0.

### 4.13 Quantification of Serum Corticosterone Using ELISA

Blood samples were collected from mice in the Control, SD, and SDR 14 d groups between 15:00 and 16:00 via unilateral ocular enucleation. Whole blood was allowed to clot at room temperature for 1 h, followed by centrifugation at 12 000 rpm and 4°C for 10 min. The supernatant serum was collected and stored at −80°C until analysis. Serum corticosterone levels were quantified using a Corticosterone ELISA Kit (ENZO, USA; Cat# ELS‐ADI‐900‐097).

### 4.14 Restraint‐Stress Protocol

To rigorously isolate the specific pathogenic effects of SD from generalized hypothalamic‐pituitary‐adrenal (HPA) axis activation, a restraint stress (RS) protocol was employed as a systemic stress control. Mice in the RS group were placed into well‐ventilated, cylindrical Plexiglas restrainers that restricted physical movement without impeding respiration. This restraint was conducted for 6 h daily (during the light phase) for five consecutive days, mirroring the duration of the SD protocol. During the restraint period, mice were deprived of food and water, which were provided ad libitum immediately upon return to their home cages. To evaluate the systemic stress response and subsequent auditory phenotype, serum samples were collected for corticosterone quantification via ELISA. Concurrently, auditory function was evaluated using ABR testing, and cochlear tissues were harvested for whole‐mount immunofluorescence to quantify CtBP2+ ribbon synapses across the apical, middle, and basal turns. All downstream assays were performed following the identical procedures described for the SD cohorts.

### 4.15 Pharmacological Interventions In Vivo

To investigate the mechanistic roles of oxidative stress and BK channel suppression, targeted pharmacological interventions were administered concurrently with the 5‐day SD paradigm. For antioxidant treatment, N‐acetylcysteine (NAC) was dissolved in sterile PBS and administered via intraperitoneal (*i.p*.) injection at a dose of 100 mg kg^−^^1^ once daily. To pharmacologically activate BK channels, NS1619 was initially dissolved in DMSO and subsequently diluted in sterile PBS to achieve a final DMSO concentration of 10% (v/v). NS1619 was administered *i.p*. at 5 mg kg^−^^1^ once daily throughout the 5‐day SD protocol. Following the completion of the 5‐day intervention, auditory function was evaluated via ABR recordings. Mice were subsequently euthanized, and cochlear tissues were harvested for the quantification of CtBP2+ presynaptic ribbons and the assessment of BK channel expression levels utilizing both whole‐mount immunofluorescence and western blotting.

### 4.16 Statistical Analysis

Zeiss LSM 880/LSM 900 was used for immunofluorescence images. Image J was used for image analysis and processing. GraphPad Prism 8.0.1 was used for significance analysis of the data and statistical graphing, choosing either one‐way ANOVA or two‐way ANOVA as appropriate, represented as mean ± standard deviation. Significance is expressed as ^*^ *p* < 0.05, ^**^ *p* < 0.01, ^***^ *p* < 0.001. Adobe Photoshop and Illustrator were used for data plotting and layout.

## Author Contributions

D.C. and Y.M. contributed equally to this work. Y.M. and R.C. conceived and designed the experiments. D.C., Y.M., L.H., X.C., F.K., Z.Q., M.G., X.W. and Y.Y. performed most of the experiments. D.C., Y.M., L.H.and P.L. contributed to critical discussion and data analysis. D.C. and Y.M. wrote the paper. D.C., Y.M., P.L. and R.C. validated the article. All authors read and approved the final manuscript.

## Conflicts of Interest

The authors declare no conflicts of interest.

## Supporting information




**Supporting File**: advs77570‐sup‐0001‐SuppMat.docx.

## Data Availability

The data that support the findings of this study are available from the corresponding author upon reasonable request.
