# Supplementary material for: Transient Sleep Deprivation Induces Persistent Auditory Neuropathy via ROS‐Initiated Neuroinflammation and BK Channel Suppression
Source: Adv Sci (Weinh). 2026 Sep 8:e77570. Online ahead of print. doi: 10.1002/advs.77570 (PMC13554391; doi:10.1002/advs.77570)
Supplement: Supplementary file 1 [file ADVS-9999-e77570-s001.docx]

Supporting Information

**Transient Sleep Deprivation Induces Persistent Auditory Neuropathy via ROS-Initiated Neuroinflammation and BK Channel Suppression**

Dan Chen, Ying Ma*, Lixuan Huang, Xin Chen, Fanliang Kong, Zhenzhou Qian, Mingyue Guan, Xinlin Wang, Yanyan Yang, Shasha Zhang*, Peng Li*, Renjie Chai*

***Correspondence to:** [**renjiec@seu.edu.cn**](mailto:renjiec@seu.edu.cn) **(R.C.);** [**yingma@seu.edu.cn**](mailto:yingma@seu.edu.cn) **(Y.M.);** [**lipeng25@mail.sysu.edu.cn**](mailto:lipeng25@mail.sysu.edu.cn) **(P.L.);** [**zhangshasha@seu.edu.cn**](mailto:zhangshasha@seu.edu.cn) **(S.Z.)**

**List of contents for supporting information**

1. **Supporting table**

- **Table 1. Basic Characteristics of Clinical Participants and Their Sleep Quality, related to Figure 1.**

2. **Supporting figures**

- **Figure S1. SD Induces a Persistent, High-Frequency Auditory Synaptopathy, related to Figure 2.**
- **Figure S2. Cochlear Synaptopathy Occurs without Hair Cell Loss nor Degeneration of SGN Peripheral Fibers, related to Figure 3.**
- **Figure S3. Restraint stress does not induce auditory dysfunction or cochlear synaptic loss, related to Figure 3.**
- **Figure S4. Transcriptomic identification of BK-channel-related alterations following sleep deprivation, related to Figure 5 and 6.**
- **Figure S5. Functional Recovery in Apical OHC is Mediated by Restored Potassium Currents, related to Figure 6.**

**1. Supporting table**

**Table S1. Basic Characteristics of Clinical Participants and Their Sleep Quality, related to Figure 1.**

|  | **Control** | **SSNHL** | ***p*-value** |
| --- | --- | --- | --- |
| **Number of participants** | 36 | 52 |  |
| **Age** | 31.06 ± 9.43 | 39.58 ± 12.72 | 0.014 |
| **Gender** |  |  |  |
| **Male** | 7 (19.44%) | 31 (59.62%) |  |
| **Female** | 29 (80.56%) | 21 (40.38%) |  |
| **PTA** | 14.25 ± 3.85 | 59.06 ±32.76 | <0.001 |
| **Total PSQI score** | 4.03 ± 1.83 | 7.94 ± 4.39 | <0.001 |
| **Subjective sleep quality** | 1.19 ± 0.58 | 1.87 ± 0.84 | <0.001 |
| **Sleep latency** | 0.81 ± 0.89 | 1.63 ± 0.87 | <0.001 |
| **Sleep duration** | 0.69 ± 0.62 | 1.10 ± 0.82 | 0.022 |
| **Sleep efficiency** | 0.56 ± 0.65 | 0.90 ± 0.80 | 0.036 |
| **Sleep disturbance** | 0.61 ± 0.49 | 0.63 ± 0.63 | 0.954 |
| **Use of sleep medication** | 0.14 ± 0.49 | 0.63 ± 0.99 | 0.015 |
| **Daytime dysfunction** | 0.39 ± 0.77 | 0.79 ± 0.98 | 0.190 |
| **VAS** | 3.61 ± 1.34 | 5.52 ± 1.80 | <0.001 |

**2. Supporting figures**

**Figure S1: SD Induces a Persistent, High-Frequency Auditory Synaptopathy, related to Figure 2. (A)** A picture depicts mice in an automated sleep deprivation system with rotating bars. **(B)** Body weight changes over the course of the experiment. **(C)** Quantification of ABR thresholds across all frequencies for all four experimental groups (Control, SD, SDR 7d, and SDR 14d). **(D)** Frequency-specific quantification of ABR wave I amplitude for Control, SD, and SDR 14d groups. Data are presented as mean ± SD. Statistical significance was determined by repeated measures ANOVA (B) or two-way ANOVA (C and D) with Tukey’s multiple comparisons test. (*n.s.*, not significant; **p* < 0.05, ***p* < 0.01, ****p* < 0.001, *****p* < 0.0001.) n = 8 mice per group.

**Figure S2: Cochlear synaptopathy occurs without hair cell loss nor degeneration of spiral ganglion neuron peripheral fibers, related to Figure 3. (A)** Representative immunofluorescence images of the organ of Corti from the apical, middle, and basal turns. Hair cells are stained with Myosin7a (red) and nuclei with DAPI (blue). Scale bar, 50 µm. **(B)** Quantification of surviving OHCs per 100µm in the apex, middle, and base of the cochlea. **(C)** Quantification of surviving IHCs per 100µm in the apex, middle, and base of the cochlea. **(D)** Representative images of spiral ganglion neuron (SGN) peripheral fibres stained for Tuj1 (green) beneath the IHCs. Scale bar, 50 µm. **(E)** Quantification of Tuj1-positive fibres per 100 µm in the apex, middle, and base. Data are presented as mean ± SD. Statistical significance was determined by one-way ANOVA with Tukey’s post-hoc test (*n.s.*, not significant; **p* < 0.05, ***p* < 0.01, ****p* < 0.001, *****p* < 0.0001).

**Figure S3. Restraint stress does not induce auditory dysfunction or cochlear synaptic loss.** **(A)** Schematic of the restraint-stress protocol. Mice were subjected to restraint stress for 6h per day for five consecutive days, followed by ABR measurements. **(B)** Serum corticosterone levels in control and restraint-stressed mice. Repeated restraint stress significantly increased serum corticosterone levels, confirming activation of the systemic stress response. **(C)** ABR thresholds at 4, 8, 12, 16, 24, and 32 kHz before and after the five-day restraint-stress protocol. No significant threshold shifts were detected at any tested frequency. **(D)** Representative confocal images of CtBP2+ presynaptic ribbons (red) beneath inner hair cells in the apical, middle, and basal turns of the cochlea. Nuclei were counterstained with DAPI (blue). Scale bar, 10 μm. **(E)** Quantification of CtBP2+ ribbons per IHC in the apical, middle, and basal cochlear turns. No significant differences were detected between the control and restraint-stress groups. Data are presented as mean ± SD; control, n = 3 mice; restraint stress, n = 4 mice. Data in (B) were analyzed using an unpaired two-tailed Student’s t-test. Data in (C) and (E) were analyzed using two-way ANOVA followed by Tukey’s multiple-comparisons test. **p* < 0.05; *n.s.*, not significant.

**Figure S4. Transcriptomic identification of BK-channel-related alterations following SD. (A)** Principal component analysis (PCA) of transcriptomic profiles from control, SD, and recovery groups.The three experimental groups showed distinct transcriptional patterns, indicating substantial global gene expression alterations induced by SD and partial restoration following recovery. **(B)** Volcano plot showing differentially expressed genes between the SD and control groups. Genes with significant differential expression are highlighted, and *Kcnma1*, encoding the pore-forming α-subunit of the large-conductance calcium-activated potassium (BK) channel, was among the SD-responsive genes identified by RNA-sequencing analysis. **(C)** Heatmap showing the expression patterns of BK channel-related genes (*Kcnma1*, *Kcnmb1*, and *Kcnmb2*) and SK channel-related genes (*Kcnn1*, *Kcnn2*, and *Kcnn3*) across control, SD, and recovery groups. Compared with SK channel genes, *Kcnma1* exhibited a more prominent SD-associated alteration and showed a tendency toward restoration after recovery, supporting its prioritization as a candidate molecular target for further investigation.

**Figure S5. Functional recovery in apical inner hair cells is mediated by restored potassium currents, related to Figure 6. (A)** Representative whole-cell patch-clamp image for recordings IHCs in the cochlear apex. **(B)** Current-voltage (I-V) relationship plots for the isolated small-conductance (SK) current density. **(C, D)** Quantification of resting membrane potential (RMP) (C) and cell membrane capacitance (Cm) (D) as key cellular health metrics. Data are presented as mean ± SD. Statistical significance was determined by one-way ANOVA with Tukey’s multiple comparisons test (*n.s.*, not significant; **p* < 0.05, ***p* < 0.01, ****p* < 0.001, *****p* < 0.0001). n = 12 cells from 6 mice per group.
